# Quantitative Computed Tomographic Clusters in C‐BIOPRED Asthma Cohort: Association with Sputum Proteomics

**DOI:** 10.1002/mco2.70471

**Published:** 2025-11-09

**Authors:** Zhenan Deng, Tingting Xia, Chenyang Lu, Xuliang Cai, Yujing Liu, Zhongmin Qiu, Xiaoyang Wei, Wei Gu, Dandan Chen, Jianping Zhao, Xiaoxia Liu, Shenghua Sun, Huaping Tang, Bei He, Shaoxi Cai, Ping Chen, Nanshan Zhong, Kian Fan Chung, Meiling Jin, Qingling Zhang, the C‐BIOPRED Consortium

**Affiliations:** ^1^ State Key Laboratory of Respiratory Disease, National Clinical Research Center for Respiratory Disease, National Center For Respiratory Medicine, Department of Pulmonary and Critical Care Medicine, Guangzhou Institute of Respiratory Health The First Affiliated Hospital of Guangzhou Medical University Guangzhou China; ^2^ Department of Radiology The First Affiliated Hospital of Guangzhou Medical University Guangzhou China; ^3^ AstraZeneca Shanghai China; ^4^ Department of Pulmonary and Critical Care Medicine, Tongji Hospital, School of Medicine Tongji University Shanghai China; ^5^ Department of Respiratory Medicine The Eighth Medical Center of PLA General Hospital Beijing China; ^6^ Department of Respiratory Medicine Nanjing First Hospital Nanjing Medical University Nanjing China; ^7^ Department of Pulmonary and Critical Care Medicine, Shenzhen Institute of Respiratory Diseases The First Affiliated Hospital (Shenzhen People's Hospital) and School of Medicine, Southern University of Science and Technology Shenzhen China; ^8^ Department of Respiratory Medicine Tongji Hospital, Tongji Medical College, Huazhong University of Science and Technology Wuhan China; ^9^ Department of Respiratory Medicine Beijing Friendship Hospital Capital Medical University Beijing China; ^10^ Department of Respiratory Medicine The Third Xiangya Hospital of Central South University Changsha China; ^11^ Department of Respiratory Medicine Qingdao Municipal Hospital Qingdao China; ^12^ Department of Respiratory Medicine Peking University Third Hospital Beijing China; ^13^ Department of Respiratory Medicine Nanfang Hospital of Southern Medical University Guangzhou China; ^14^ Department of Respiratory Medicine General Hospital of Northern Theater Command Shenyang China; ^15^ Guangzhou National Laboratory Bioland Guangzhou China; ^16^ National Heart and Lung Institute Imperial College London London UK; ^17^ Royal Brompton and Harefield Hospital, Guy's St Thomas NHS Foundation Trust London UK; ^18^ Department of Respiratory Medicine Zhongshan Hospital Shanghai China

**Keywords:** airflow obstruction, asthma, airway remodeling, high‐resolution computed tomography, proteomics

## Abstract

Severe asthma exhibits heterogeneity in airflow obstruction, driven by airway remodeling and air trapping, which can be noninvasively assessed via quantitative computed tomography (qCT). This study aimed to identify asthma phenotypes by clustering qCT measurements of airway dimensions, lung volumes, and densitometry, and to elucidate the underlying molecular pathways through sputum proteomics. We applied consensus clustering to qCT data from 239 asthma patients (severe and mild/moderate) and 68 healthy controls from the Chinese C‐BIOPRED cohort. Four distinct qCT clusters emerged: cluster 1, characterized by luminal dilation, severe air trapping, and reduced lung density; cluster 2, with thickened airway walls and luminal narrowing without air trapping; cluster 3, showing mild luminal dilation, preserved lung volumes, and optimal spirometry; and cluster 4, featuring airway wall thickening, luminal narrowing, severe air trapping, and profound airflow obstruction. Sputum eosinophilia was elevated in clusters 1 and 4. Proteomics revealed upregulated pathways in apoptosis execution and cornified envelope formation in cluster 1, while clusters 2 and 4 exhibited enhanced complement activation, fibrin formation, plasma lipoprotein assembly, and insulin‐like growth factor (IGF) transport regulation. These findings delineate qCT‐derived phenotypes and their associated underlying mechanisms of airway remodeling and airflow obstruction in severe asthma.

## Graphical abstract

Using quantitative computed tomography (qCT) clustering in the C‐BIOPRED asthma cohort of 239 patients, four distinct phenotypes were identified based on airway dimensions, lung volumes, and densitometry, revealing variations in airway remodeling, air trapping, and airflow obstruction compared with healthy controls. Cluster 1 showed airway dilation with severe air trapping and reduced lung density; cluster 2 featured thickened airway walls and luminal narrowing without air trapping; cluster 3 displayed mild airway dilation, preserved lung volumes, and optimal spirometry; and cluster 4 exhibited airway wall thickening, luminal narrowing, severe air trapping, and profound airflow obstruction. Sputum eosinophilia was elevated in clusters 1 and 4. Sputum proteomics linked cluster 1 to upregulated apoptotic execution and cornified envelope formation pathways, while clusters 2 and 4 showed enhanced complement activation, fibrin formation, plasma lipoprotein assembly, and IGF transport regulation, providing insights into underlying molecular mechanisms of severe asthma heterogeneity.

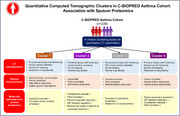

## 1 Introduction

Asthma remains one of the most prevalent chronic respiratory diseases worldwide, affecting over 300 million individuals and imposing a significant burden on healthcare systems due to its associated morbidity and mortality [1]. A subset of patients, approximately 5%–10% of the asthmatic population, experiences severe asthma, defined by persistent symptoms, frequent exacerbations, and evidence of airflow obstruction despite maximal treatment with high‐dose inhaled corticosteroids and additional controllers [2].

The heterogeneity of severe asthma is well‐recognized, manifesting in diverse clinical phenotypes driven by distinct endotypes, such as eosinophilic, neutrophilic, or paucigranulocytic inflammation [3]. Airflow obstruction results from processes such as mucus plugs, inflammatory exudates, airway wall thickening (from smooth muscle hypertrophy and subepithelial fibrosis), or changes in airway lumen size (dilatation or narrowing). These structural changes, collectively termed airway remodeling, contribute to fixed airflow limitation and reduced lung function, as measured by spirometric indices like forced expiratory volume in one second (FEV1). Understanding these structural underpinnings is crucial for identifying targeted interventions that address the root causes rather than merely alleviating symptoms [4, 5].

Noninvasive imaging techniques have revolutionized the assessment of airway and parenchymal changes in asthma, with high‐resolution computed tomography (HRCT) emerging as a key tool for visualizing the tracheobronchial tree and lung parenchyma [6]. Quantitative CT (qCT) extends beyond qualitative observations by providing precise measurements of airway dimensions (e.g., wall thickness and luminal area) and lung densitometry (e.g., air trapping and emphysema‐like changes) [7, 8]. qCT metrics, such as increased wall area percentage (WA%) and air trapping indices like percent voxels less than −856 HU on expiratory phase (VI‐856%), correlate strongly with clinical severity, FEV1 reduction, bronchodilator responsiveness, and asthma control scores [9, 10, 11, 12]. Furthermore, qCT has revealed associations between air trapping and neutrophilic inflammation, as well as between airway remodeling and T2 biomarkers like fractional exhaled nitric oxide (FeNO) [8, 13].

Unsupervised clustering of qCT parameters has revealed phenotypes with varying airway wall thickening, luminal changes, and air trapping. These often align with disease severity and inflammation [14, 15, 16]. However, ethnic differences in asthma presentation, such as higher eosinophilia in Asian populations, suggest that qCT clusters may vary geographically, warranting studies in diverse cohorts.

In this study, we utilized qCT measurements from lung HRCT scans of China's first severe asthma multicenter cohort (C‐BIOPRED) [17] to obtain new clusters. We compared these clusters to prior non‐Chinese cohorts [14, 16, 18]. Furthermore, we determined whether the proteome of sputum samples obtained from these asthmatics can indicate the potential mechanisms underlying these clusters. Our data indicate that proximal airway wall thickening and airway wall narrowing may be associated with the regulation of insulin‐like growth factor (IGF) transport, plasma lipoprotein assembly, and the activation of the complement cascade and fibrin formation.

## 2 Results

### 2.1 Clinical Characteristics and Quantitative CT Parameters of Participants

Table 1 summarizes the clinical characteristics of the asthma groups. Asthmatic patients were older, had higher body mass index (BMI), and elevated type 2 biomarkers than healthy controls (HC). These traits were more pronounced in severe asthmatics. The ex‐smokers with severe asthma (SSA) group showed the worst airflow obstruction compared with the nonsmoking severe asthma (NSA) and mild/moderate asthma (MMA) groups.

**TABLE 1:** Clinical characteristics of asthma patients and healthy subjects.

Characteristics	Nonsmoking severe asthma (NSA, *n *= 132)	Smokers and ex‐smokers with severe asthma (SSA, *n *= 49)	Mild/moderate asthma (MMA, *n *= 58)	Healthy controls (HC, *n *= 68)	*p*‐value
Age (years)	54.0 (11.25)	58.0 (10.0)	45.5 (21.5)	25.0 (14.75)	<0.001
Gender (male %)	42 (32)	48 (98)	29 (50)	26 (38)	<0.001
BMI (kg/m^2^)	23.61 (4.01)	24.09 (3.23)	23.14 (3.96)	21.68 (4.04)	0.002
BSA (m^2^)	1.59 (0.24)	1.75 (0.16)	1.67 (0.26)	1.57 (0.25)	<0.001
Age at diagnosis (years)	46.48 (19.08)	49.50 (19.70)	36.58 (19.76)	NA	<0.001
ACQ5	1.40 (1.60)	2.00 (1.00)	0.90 (1.20)	NA	<0.001
AQLQ	4.50 ± 1.17	4.57 ± 1.08	5.29 ± 0.90	NA	<0.001
Exacerbations in previous year	1.41 ± 1.85	1.29 ± 1.44	0.33 ± 0.55	NA	<0.001
Pre‐bronchodilator FEV1 (% predicted)	70.02 (27.49)	57.04 (30.90)	81.00 (27.21)	NA	<0.001
Pre‐bronchodilator FEV1/FVC (%)	62.09 (20.27)	56.13 (22.89)	65.59 (20.94)	NA	0.01
Blood eosinophils (×10^9^/L)	0.25 (0.32)	0.28 (0.30)	0.24 (0.17)	0.09 (0.10)	<0.001
FeNO (ppb)	36.0 (44.0)	31.0 (47.0)	30.0 (27.0)	15.0 (8.0)	<0.001
Total IgE (KU/mL)	194.00 (354.05)	183.00 (246.90)	178.50 (381.77)	28.95 (59.38)	<0.001
ECP	7.89 (10.81)	8.30 (9.44)	8.31 (10.90)	3.29 (3.63)	<0.001
Sputum eosinophils (%)	16.97(41.69)	12.95(31.07)	4.98 (27.77)	0.63 (0.97)	<0.001
Sputum macrophages (%)	9.36 (24.56)	8.39 (12.94)	14.89 (38.66)	35.50 (57.41)	0.03
Sputum neutrophils (%)	54.59 (59.66)	66.27 (50.14)	52.01(52.71)	52.93 (57.70)	0.55
Atopy (%)	68 (53)	26 (54)	18(33)	49 (75)	0.00077
OCS maintenance (%)	9 (6.8)	5 (10)	0 (0)	0 (0)	0.011

*Note*: Data are shown as median (interquartile range [IQR]), unless as *n* (%). *p‐*values were calculated using the Kruskal–Wallis rank sum test for continuous variables and Fisher test for categorical variables. Atopy (above normal) means the subjects have at least one allergen above normal.

Abbreviations: ACQ5, asthma control questionnaire; AQLQ, asthma quality of life questionnaire; BMI, body mass index; BSA, body surface area; ECP, eosinophil cationic protein; FeNO, fraction of nitric oxide in Expiratoryd breath; Max, maximum; Min, minimum; *n*, number of subjects included in the analysis; NA, not applicable; OCS, oral corticosteroid; SD, standard deviation.

For qCT airway parameters, mean lumen area/body surface area (LA/BSA) was lower in NSA than in MMA and healthy controls (HC). Mean wall area percentage (WA%) was higher in NSA than HC, but differences were not significant. Pi10WA (wall area of a hypothetical airway with 10 mm internal perimeter) was elevated in both severe asthma groups versus MMA and HC, highest in SSA. Other airway parameters showed no significant differences.

qCT densitometry revealed higher emphysema and air trapping measures in severe asthmatics: expiratory lung volume (expiratory LV), expiratory mean lung density (expiratory MLD), mean lung density expiratory/inspiratory ratio (MLD E/I), inspiratory percent voxels <−950 HU (inspiratory VI‐950%), and expiratory air trapping (expiratory VI‐856%). SSA had the highest of these values (Table 2).

**TABLE 2:** Quantitative airway CT parameters in asthma patients and healthy subjects.

Characteristics	Nonsmoking severe asthma (NSA, *n *= 132)	Smokers and ex‐smokers with severe asthma (SSA, *n *= 49)	Mild/moderate asthma (MMA, *n *= 58)	Healthy controls (HC, *n *= 68)	*p*‐value
Mean LA/BSA (mm^2^/m^2^)	9.04 (3.53)	9.84 (3.60)	9.82 (3.09)	9.96 (3.53)	0.069
Mean WA%	64.81 (3.87)	64.67 (4.93)	63.85 (4.24)	63.72 (3.51)	0.12
Mean Pi10WA (mm^2^)	17.18 (3.59)	18.23 (4.04)	16.73 (3.33)	15.92 (3.01)	<0.001
Expiratory LV(L)	2.94 (1.17)	4.14 (1.96)	2.84 (1.37)	2.57 (0.90)	<0.001
Expiratory MLD (HU)	−747.91 (76.06)	−773.52 (87.24)	−742.28 (68.35)	−721.46 (49.47)	<0.001
Expiratory VI‐856 (%)	24.69 (29.42)	33.50 (36.12)	20.52 (22.37)	10.06 (13.05)	<0.001
MLD E/I	0.91 (0.07)	0.92 (0.07)	0.89 (0.08)	0.87 (0.08)	<0.001
Inspiratory VI‐950 (%)	4.40 (9.25)	5.58 (9.02)	3.84 (6.96)	1.58 (2.71)	<0.001

*Note*: Data are shown as median (interquartile range [IQR]), unless as *n* (%). *p*‐values were calculated using the Kruskal–Wallis rank sum test for continuous variables and the Fisher test for categorical variables.

Abbreviations: Max, maximum; Min, minimum; *n*, number of subjects included in the analysis; NA, not applicable; SD, standard deviation.

### 2.2 Correlation Between qCT Parameters and Clinical Indicators, and Their Diagnostic Efficacy

Correlation analysis (Table S4) revealed a positive association between airway wall thickness (Pi10WA) and sputum neutrophils. Measures of lung density, such as expiratory MLD and MLD E/I, were inversely and positively correlated with FeNO and sputum eosinophils, respectively—both biomarkers of type 2 inflammation. Additionally, sputum macrophages (%) negatively correlated with expiratory LV, MLD E/I, expiratory VI‐856(%), and inspiratory VI‐950(%), but positively with expiratory MLD.

Receiver operating characteristic (ROC) curves were used to assess the performance of qCT parameters in distinguishing disease severity (severe vs. mild/moderate asthma, Figure S2) and endotype (eosinophilic vs. non‐eosinophilic asthma, Figure S3). MLD E/I had the highest AUCs: 0.63 for severity and 0.61 for endotype.

### 2.3 Cluster Analysis

**FIGURE 1:**
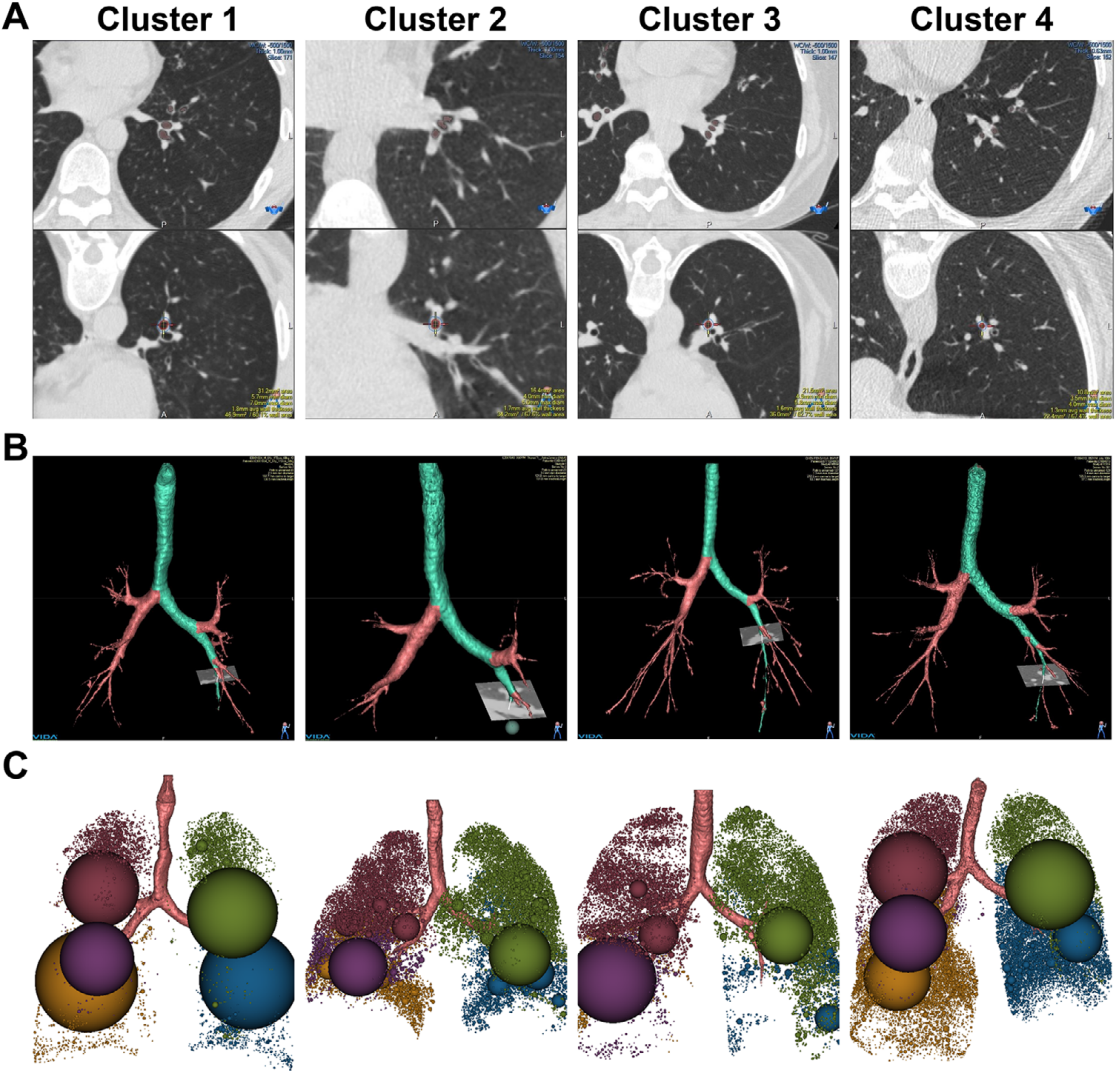
HRCT imaging and analysis of airway and emphysematous features across four clusters. (A) Upper row displays cross‐sectional HRCT images of the third‐order bronchus (LB10) for luminal area assessment; lower row shows airway wall thickness measurements. (B) Tracheobronchial tree reconstructed using VIDA Workstation (version 1.2). (C) Density mask method quantifies air trapping and emphysematous lesions, with color‐coded spheres indicating low attenuation areas (<−856 HU) by pulmonary lobe.

We performed principal component and cluster analyses on qCT data from asthmatic patients using 8 parameters. The consensus matrix indicated *n* = 4 as optimal (Figure S1). Figure 1 presents representative CT images from the four clusters. Panels A–C depict airway remodeling, tracheobronchial 3D reconstruction, and emphysematous/air trapping areas.

**FIGURE 2:**
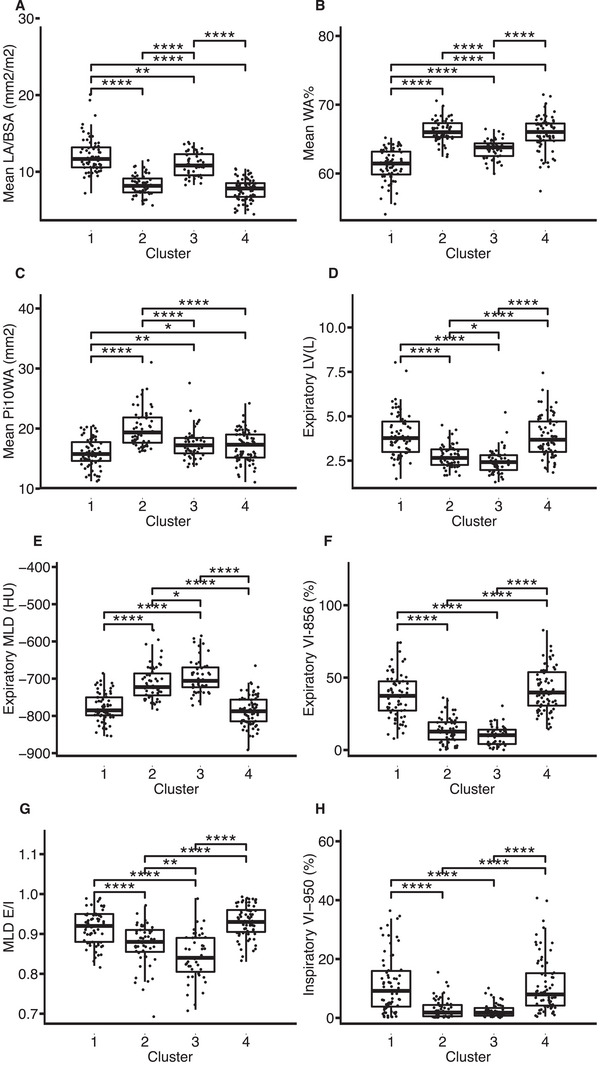
Quantitative CT metrics across four qCT clusters. (A) Mean LA/BSA (mm^2^/m^2^). (B) Mean WA%. (C) Mean Pi10WA (mm^2^). (D) Expiratory LV(L). (E) Expiratory MLD(HU). (F) Expiratory VI‐856(%). (G) MLD E/I. (H) Inspiratory VI‐950(%). Data shown as median (interquartile range). *p < 0.05, **p < 0.01, ***p < 0.001, ****p < 0.0001.

Clusters were defined by proximal airway thickening, luminal narrowing, and air trapping (Table S3). Compared with HC, the mean LA/BSA was lower in clusters 2 and 4, higher in clusters 1 and 3 (cluster 1 > 3) (Figure 2A). Mean WA% and Pi10WA were elevated in clusters 2, 3, and 4 (Figure 2B, C). Expiratory LV, MLD E/I, expiratory VI‐856(%), and inspiratory VI‐950(%) were higher in clusters 1 and 4; expiratory MLD was lower (Figure 2D–H).

In summary, the four clusters exhibited distinct radiographic phenotypes: Cluster 1 showed severe air trapping, luminal dilatation, and reduced lung density without wall thickening. Cluster 2 had wall thickening and luminal narrowing without air trapping or density reduction. Cluster 3 featured mild luminal dilatation without wall thickening, air trapping, or density reduction. Cluster 4 combined wall thickening, luminal narrowing, severe air trapping, and reduced density.

### 2.4 Clinical Characteristics of qCT Clusters

Table 3 details demographics and clinical traits. Cluster 1 had more males (64%) and smokers (30%). Cluster 2 had the highest BMI (24.77). Cluster 3 had the most females (66%) and the fewest smokers (13%). Severe asthma proportions and exacerbations were similar across clusters. Asthma control questionnaire (ACQ5) was highest, and asthma quality of life questionnaire (AQLQ) was lowest in cluster 3. Prebronchodilator FEV1 (% predicted) was lowest in cluster 4. Cluster 1 had the highest sputum eosinophil %, but not significantly different. Other type 2 inflammation indicators, such as blood eosinophil counts, FeNO, serum total immunoglobulin E (IgE) and eosinophil cationic protein (ECP) concentrations, and atopy, were similar across the four clusters.

**TABLE 3:** Clinical characteristics of the four clusters.

Characteristic	Cluster 1 (*n* = 66)	Cluster 2 (*n* = 55)	Cluster 3 (*n* = 47)	Cluster 4 (*n* = 71)	*p*‐value
Age (years)	54.00 (16.00)	54.00 (10.50)	52.00 (21.50)	55.00 (15.00)	NS
Gender (male %)	42 (64)	25 (45)	16 (34)	36 (51)	0.017
BMI (kg/m^2^)	23.01 (3.81)	24.77 (4.49)	23.22 (3.86)	23.66 (4.06)	0.0031
BSA (m^2^)	1.66 (0.22)	1.73 (0.28)	1.63 (0.27)	1.64 (0.23)	NS
Age at diagnosis (years)	46.31 (22.31)	46.31 (21.03)	41.70 (23.82)	45.74 (19.32)	NS
Smoking (%)	20 (30)	9 (16)	6 (13)	14 (20)	NS
Severity (%)	46 (70)	42 (76)	36 (77)	57 (80)	NS
ACQ5	1.60 (1.20)	1.60 (1.60)	0.80 (1.40)	1.60 (1.20)	<0.001
AQLQ	4.63 ± 1.12	4.60 ± 1.18	5.25 ± 1.08	4.49±1.06	0.0026
Exacerbations in previous year	1.39 ± 1.92	0.94 ± 1.16	0.80 ± 0.93	1.23±1.88	NS
Pre‐bronchodilator FEV1 (% predicted)	73.00 (34.80)	72.68 (23.80)	79.38 (25.57)	56.52 (24.27)	<0.001
Pre‐bronchodilator FEV1/FVC (%)	62.82 (17.65)	61.32 (19.42)	68.67 (12.86)	52.21 (20.22)	<0.001
Blood eosinophils (×10^9^/L)	0.24 (0.26)	0.26 (0.18)	0.23 (0.23)	0.24 (0.38)	NS
Blood neutrophils (×10^9^/L)	3.78 (1.53)	3.79 (1.61)	3.82 (2.45)	3.53 (1.64)	NS
FeNO (ppb)	41.00 (55.25)	36.00 (31.00)	28.00 (29.00)	35.00 (48.50)	NS
Total IgE (KU/mL)	162.00 (318.50)	179.00 (348.25)	254.00 (435.95)	160.00 (356.90)	NS
ECP	7.88 (10.68)	8.30 (8.54)	7.36 (7.07)	8.57 (14.93)	NS
Sputum eosinophils (%)	26.53 (56.70)	9.83 (18.66)	5.12 (36.44)	15.60 (36.28)	NS
Sputum neutrophils (%)	46.13 (58.05)	60.96 (28.53)	45.60 (62.12)	59.29 (55.56)	NS
Sputum macrophages (%)	7.03 (15.47)	18.19 (25.76)	10.73 (36.67)	9.44 (14.63)	NS
Atopy (%)	33 (53)	25 (46)	18 (39)	36 (53)	NS
OCS maintenance (%)	7 (11)	5 (9.1)	1(2.1)	6 (8.5)	NS

*Note*: Data are shown as median (interquartile range [IQR]), unless as *n* (%). *p*‐values were calculated using Kruskal–Wallis rank sum test for continuous variables and Fisher test for categorical variables. Atopy (above normal) means the subjects have at least one allergen above normal.

Abbreviations: ACQ5, asthma control questionnaire; AQLQ, asthma quality of life questionnaire; BMI, body mass index; BSA, body surface area; ECP, eosinophil cationic protein; EOS, eosinophilic granulocyte; FeNO, fraction of nitric oxide in exhaled breath; Max, maximum; Min, minimum; n, number of subjects included in the analysis; NA, not applicable; OCS, oral corticosteroid; SD, standard deviation.

### 2.5 Validation of Identified Clusters Using Decision Tree Analysis

**FIGURE 3:**
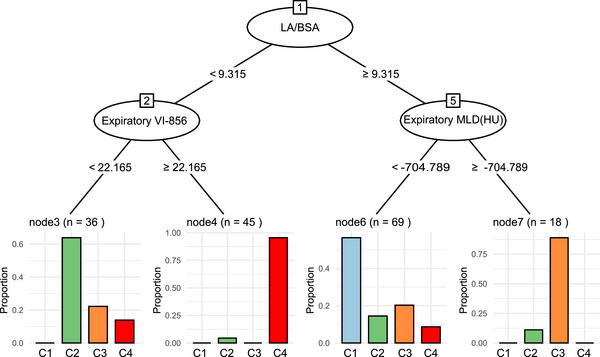
Decision tree for cluster classification using qCT variables. Using three variables, LA/BSA, expiratory VI‐856(%), and expiratory MLD(HU), subjects were classified into four clusters.

To predict the accuracy of cluster assignment in patients, we developed a tree diagram. Data from 239 patients with asthma were randomly divided into a training sample (70%) and a validation sample (30%). A conditional inference tree analysis was performed in the training sample using qCT variables to assess the classification of patients. Using three variables, LA/BSA, expiratory VI‐856(%), and expiratory MLD, patients were assigned to the appropriate cluster (Figure 3). In the validation sample, 88.7% of patients could be assigned to the correct cluster through the tree analysis scheme. This indicates that a simple method for phenotyping of asthma could be based on these qCT variables.

### 2.6 Molecular Pathways of qCT Clusters

**FIGURE 4:**
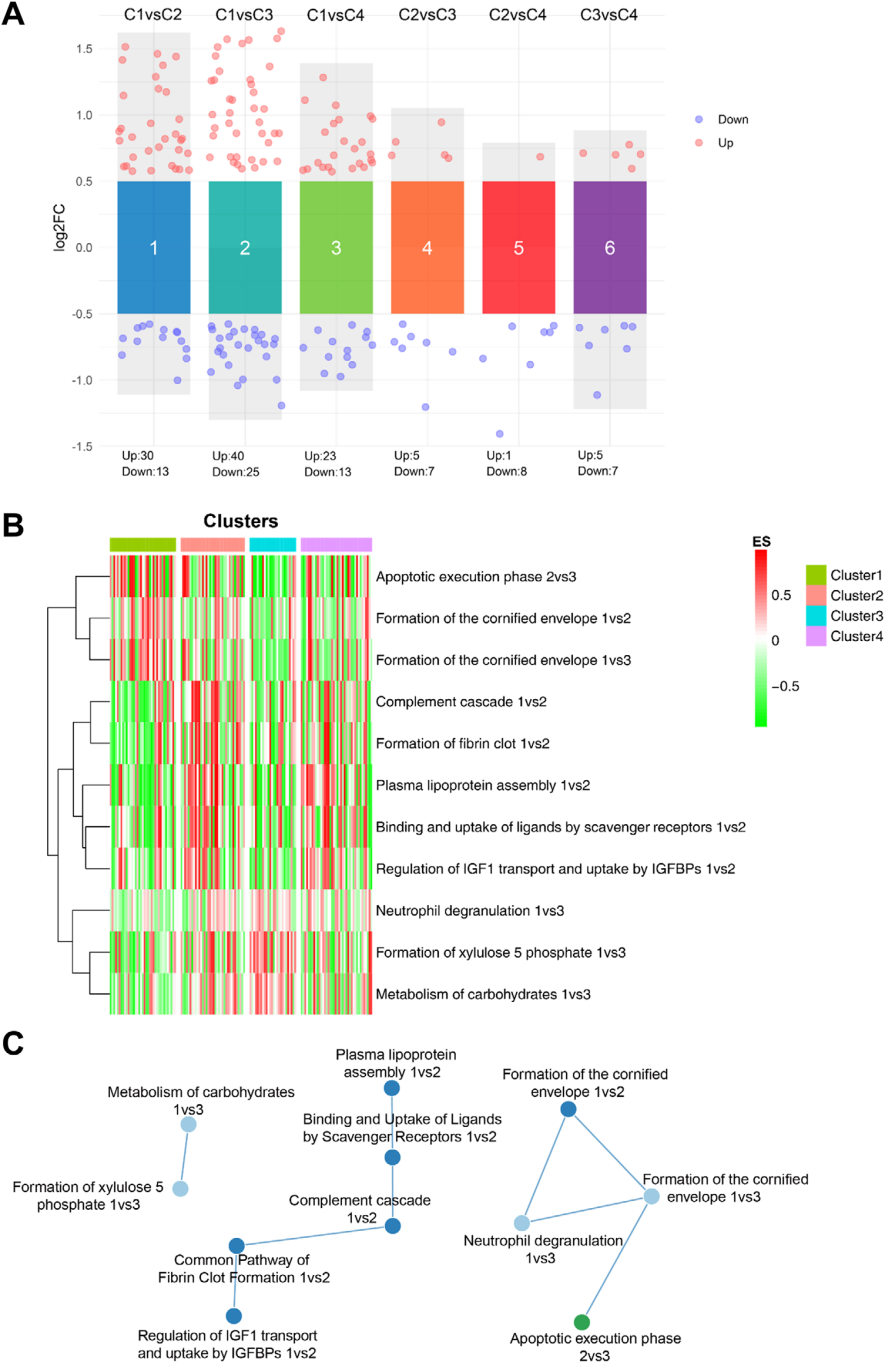
Proteomic profiling of sputum supernatant across clusters. (A) The volcano plot illustrates differentially expressed proteins between clusters. (B) Enrichment scores of 11 proteomic pathways (rows) across subjects (columns) by cluster, with signatures listed in Table S6. (C) Enrichment maps show pathway networks connected by shared genes.

**FIGURE 5:**
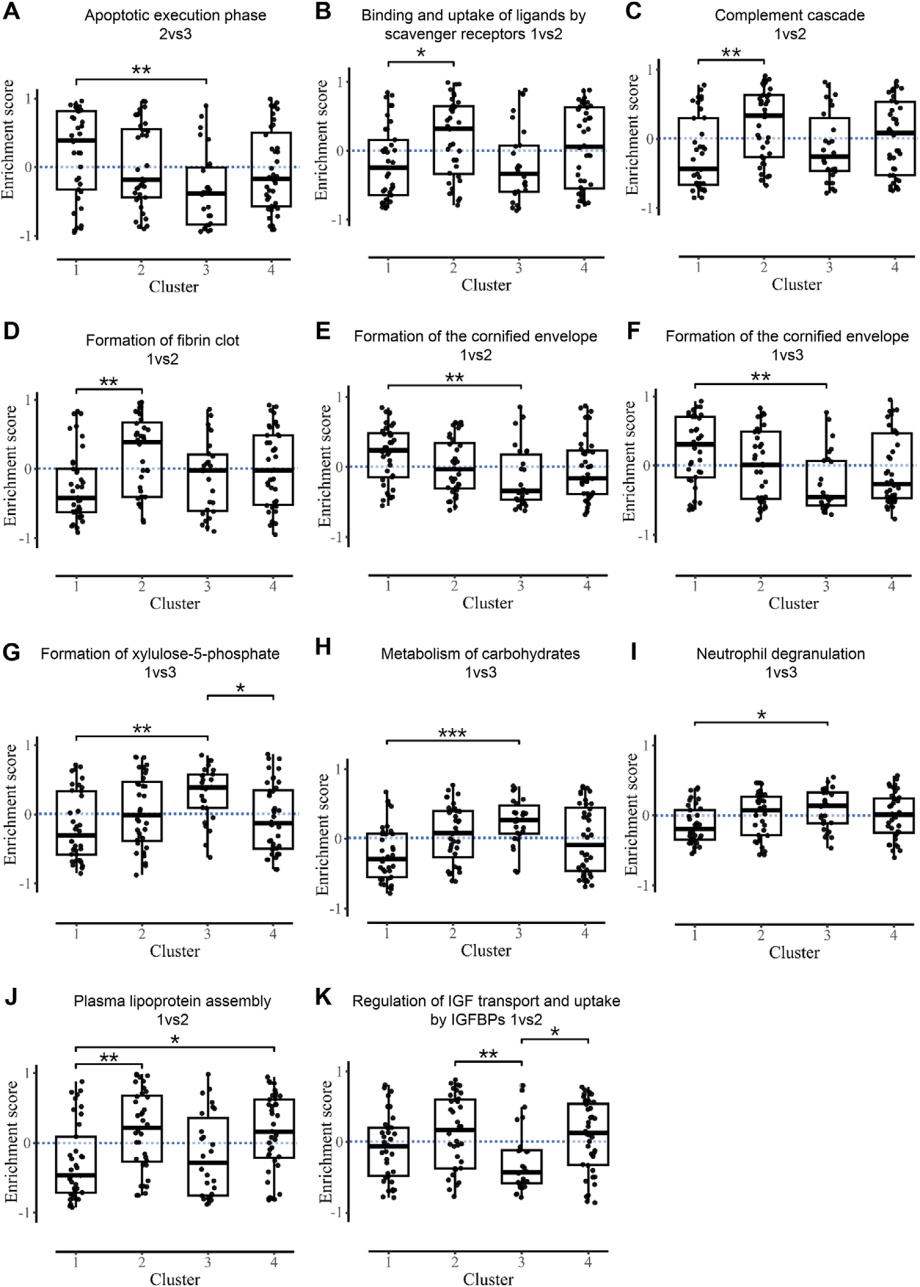
Gene set variation analysis (GSVA) scores of differentially expressed pathways. (A) Apoptotic execution phase 2vs3. (B) Binding and uptake of ligands by scavenger receptors 1vs2. (C) Complement cascade 1vs2. (D) Formation of the cornified envelope 1vs2. (E) Formation of the cornified envelope 1vs3. (F) Formation of fibrin clot 1vs2. (G) Formation of xylulose‐5‐phosphate 1vs3. (H) Metabolism of carbohydrates 1vs3. (I) Neutrophil degranulation 1vs3. (J) Plasma lipoprotein assembly 1vs2. (K) Regulation of IGF transport and uptake by IGFBPs 1vs2. Data shown as median (interquartile range). *p < 0.05, **p < 0.01, ***p < 0.001.

We analyzed sputum supernatant proteomics across clusters versus HC. Binary comparisons yielded 43, 65, 36, 12, 9, and 12 differentially expressed proteins (DEPs) (Figure 4A, Table S6). Reactome gene set variation analysis (GSVA) was performed to visualize the pathway enrichment scores and molecular signatures across the clusters. As shown in Table S5 and Figures 4B and 5, 11 pathways were identified with adjusted *p*‐values <0.05 across clusters. Specifically, cluster 1 was characterized by significant upregulation in the apoptotic execution phase and formation of the cornified envelope pathways; cluster 2 by upregulated pathways of binding and uptake of ligands by scavenger receptors, the complement cascade, formation of fibrin, plasma lipoprotein assembly, and regulation of IGF transport and uptake by IGFBPs; cluster 3 by upregulation of the formation of xylulose‐5‐phosphate and the metabolism of carbohydrate pathways; and cluster 4 by very prominent upregulation of plasma lipoprotein assembly and regulation of IGF transport and uptake by IGFBPs.

Enrichment maps showed pathway interconnections (Figure 4C). The complement cascade hub is linked to fibrin clot (via PROS1, SERPIND1) and scavenger receptors (via IGKV1.5, IGLV1.47, IGHV4.34). It extended to neutrophil degranulation, cornified envelope, and apoptotic execution (via KRT1, DSP, DSG3, SPRR3). IGF regulation is connected to fibrin via SERPIND1. Plasma lipoprotein assembly linked to scavenger receptors via APOE. Carbohydrate metabolism and xylulose‐5‐phosphate are paired via AKR1A1, SORD. These networks highlight integrated inflammation, barrier function, and lipid/protein regulation.

### 2.7 Associations of Molecular Pathways With qCT Parameters and Clinical Indicators

Figure S4 displays the correlations between various sputum proteomics pathways and qCT, lung function, and sputum inflammation parameters. Complement cascade and fibrin formation positively correlated with lung function and negatively with cornified envelope. Expiratory VI‐856(%) and Inspiratory VI‐950(%) positively correlated with complement and fibrin, and negatively with cornified envelope and apoptosis. Complement, fibrin, lipoprotein assembly, and IGF regulation positively correlated with WA% and negatively with LA/BSA.

Most pathways positively correlated with sputum eosinophils (%). Cornified envelope and apoptosis positively correlated with sputum neutrophils (%).

## 3 Discussion

By clustering on the qCT measurements of HRCT scans of 239 patients with mild–moderate and severe asthma, we defined four clusters with varying airway wall thickness, lumen size, air trapping, and airflow obstruction, compared with healthy controls. Thus, cluster 2 showed narrowed lumens and thickened walls. Clusters 1 and 4 both had severe air trapping and reduced lung density. However, cluster 4 also had severe wall thickening and luminal narrowing, while cluster 1 had luminal dilatation. Cluster 3 showed mild luminal dilatation with preserved wall thickness, volumes, and density.

One could surmise that the changes in airway wall thickening as a reflection of an airway remodeling process could account for the degree of airflow obstruction seen in cluster 4. This is supported by the finding that clusters 2 and 3, with minimal or no airway abnormalities, had little evidence of airflow obstruction. The patients in clusters 1 and 4 represent the extremes of severe airflow obstruction in the presence of similar degrees of eosinophilic inflammation. These features underscore a spectrum where parenchymal air trapping drives obstruction in eosinophilic‐dominant clusters (1 and 4), while proximal remodeling predominates in others (2 and 4), providing a framework for targeted phenotyping.

Our clusters bear similarities to those already reported [14, 15, 16, 18]. In Zhang et al.’s [15] study, three clusters of uncontrolled asthma were graded according to the severity of air trapping into mild, moderate, and severe, with small differences in airway wall thickness and diameter. Choi et al. [16] showed clusters based on airway wall diameter and thickness, with or without air trapping. Gupta et al. [14] also described two clusters with severe air trapping associated with airway wall thickening plus lumen changes, with a third cluster with moderate air trapping without many proximal airway remodeling changes. Moreover, in the recently described qCT measurement of the European severe asthma cohort, U‐BIOPRED, a cluster of severe airflow obstruction was present, similar to our cluster 4 [18]. Therefore, a recurrent phenotype is severe obstruction with air trapping, wall thickening, and luminal narrowing. This cluster is associated with high sputum eosinophilia as reported previously [14, 15, 18]. In the study by Choi et al. [16], this cluster was linked with both sputum neutrophilia and eosinophilia.

Clustering according to qCT provides a different type of classification from that according to the severity of asthma. Nonsmoking severe and mild/moderate asthma showed little qCT difference, except for higher MLD E/I in severe. Smoking severe asthma differed most: increased wall size, air trapping, hyperinflation, and density reduction. These may be linked to the effects of cigarette‐smoking itself in causing airflow obstruction with lung hyperinflation and air trapping, while the possibility of emphysema cannot be excluded. Interestingly, the degree of airway wall thickness as measured by Pi10WA was positively correlated with sputum neutrophils, while measures of lung density (expiratory MLD and MLD E/I) were inversely and positively correlated with FeNO and sputum eosinophils, respectively, both being biomarkers of type 2 inflammation. Of interest, the distribution of current and ex‐smoker patients amongst the four clusters was not significantly different, with the highest incidence of 30% in cluster 1. These smoking patients with eosinophilic asthma represent an already‐defined phenotype of asthma both in U‐BIOPRED [19] and in a Japanese asthma cohort [20].

The other important finding of our analysis is the association of specific molecular pathways for clusters through sputum proteomic analysis. In the most severely obstructed cluster 4 with airway wall thickening and luminal narrowing, lung density reduction, and severe air trapping, there was an upregulation of plasma lipoprotein assembly and regulation of IGF transport and uptake by IGFBPs. These two pathways were also upregulated in cluster 2, which also showed airway wall thickness and airway luminal narrowing, but with only mild airflow obstruction. Serum lipoproteins, such as triglycerides, low‐density lipoproteins, cholesterol, and apolipoprotein B, have been associated with more severe airflow obstruction and serum apolipoprotein B with FeNO and blood eosinophils in atopic asthma [21, 22], suggesting that these lipoproteins may be involved in airway wall remodeling. Apolipoprotein B, linked to type 2 inflammation, may enhance systemic inflammatory conditions [23], but this has not been studied in asthma. IGF‐IGFBPs complexes modulate IGF availability, and IGF‐1 plays a critical role in enhancing subepithelial fibrosis, airway inflammation, airway hyperresponsiveness, and airway smooth muscle hyperplasia [24]. In addition, IGF‐1 is a potent mitogen for airway smooth muscle cells [25] and myofibroblasts [26], both important components of airway wall remodeling.

The complement pathway was also increased in cluster 2, which may be related to the activation of complement C5a, which has been shown to induce chemotaxis and recruitment of eosinophils in asthma [27, 28] and exacerbate eosinophilic inflammation [29]. In addition, cluster 2 was also associated with fibrin formation. Fibrin products formed through the procoagulant activity and impaired fibrinolysis [30] lead to fibrin deposition in the distal airways and alveoli of an allergic mouse model to cause airway hyperresponsiveness [31]. In segmental airway bronchoprovocation with allergen in allergic asthmatics, fibrin formation and coagulation, and apolipoprotein pathways were induced [32]. Notably, U‐BIOPRED blood proteomics showed complement and IGF upregulation in a cluster like our cluster 2 (mild obstruction, wall thickening, no air trapping) [18].

Finally, cluster 1 (severe trapping, density reduction, luminal dilatation, mild obstruction) upregulated apoptosis execution and cornified envelope formation. Apoptosis activates caspases, leading to phagocytosis [33]. Increased epithelial apoptosis may enhance airway remodeling [34], possibly triggering squamous metaplasia via cornified envelope—a barrier process. However, these potential pathways need to be confirmed in the morphological examination of the airways of this cluster.

Our study has some limitations. First, we were limited in the various omics analyses that we could perform, and were only able to run the sputum proteomics. However, sputum proteomics is a good reflection of the inflammatory events occurring within the airway wall and lumen. Second, we were not able to validate our clusters in another similar cohort, but our results bear resemblance to previously described qCT cohorts. Finally, these associations of the pathways involving the complement cascade and IGF transport on the airway wall remodeling process need to be confirmed by wet lab experiments.

Furthermore, the integration of artificial intelligence (AI) offers promising avenues for advancing asthma management based on our findings [35]. AI algorithms can automate the identification of qCT‐derived clusters with high accuracy, then link to underlying omics‐related molecular pathways for precise diagnosis. This may facilitate personalized treatment, such as targeting IGF or complement pathways with biologics, improving outcomes in heterogeneous severe asthma populations. Future studies should validate AI models in diverse cohorts to realize this potential.

In summary, clustering of qCT measurements of airways and lungs yields phenotypes that link up with mechanisms of airway remodeling and airflow obstruction in severe asthma. qCT measurements should be an important part of the assessment of patients with severe asthma.

## 4 Material and Methods

### 4.1 Subjects

239 patients with asthma, consisting of nonsmoking severe asthma (NSA, *n* = 132), smokers and ex‐smokers with severe asthma (SSA, *n* = 49), nonsmoking mild/moderate asthma (MMA, *n* = 58), and nonsmoking healthy control subjects (HC, *n* = 68) who had an HRCT scan performed were included in this study. These patients were part of the C‐BIOPRED cohort recruited from 15 provinces in China between 2015 and 2018, as previously described [36]. Classification of asthma severity was based on international ERS/ATS guidelines for severe asthma [2]. The study was approved by the ethics committees of each recruiting center. All participants gave written informed consent for their participation in the study. All HRCT scans and induced sputum samples were collected concurrently or within 24 h of each other for each patient during the baseline visit. The patients were studied in a stable phase with no exacerbations or acute respiratory infections, and had no adjustments to their asthma medication within the prior 4 weeks, as per the C‐BIOPRED protocol [37].

### 4.2 CT Scanning Equipment and Parameters

No less than a 16‐row CT scanner was used to image the subjects. The scanner was calibrated each day with the following scanning parameters: tube voltage: ≤120 kV, tube current: ≤30 mAs, image reconstructed layer thickness: ≤1.0 mm, image reconstructed interval: ≤0.8 mm, and acquisition matrix of 512 × 512. A soft tissue smooth algorithm was used to reconstruct images. Subjects were briefly trained to take a deep breath, hold it, and expire maximally. They were all trained to perform the above manoeuvre satisfactorily prior to inclusion in the study. Two scans were obtained, one at full inspiration (near total lung capacity) and the other at the deep end of expiration (near residual volume).

### 4.3 Quantitative CT Scan Analysis

A fully automated software, VIDA Workstation, software version 1.2 (VIDA Diagnostics), was used for quantitative airway and lung analysis [38, 39]. The apical segmental bronchus of the right upper lobe (RB1), the posterior basal segmental bronchus of the right lower lobe (RB10), and the posterior basal segmental bronchus of the left lower lobe (LB10) were selected as the target bronchial tubes for detailed analysis.

The airway parameters [lumen area (LA), wall area (WA), total area (TA), and percentage of wall area (WA%)] were recorded at the midpoint of the third segmented bronchus, and the length was determined using a digital caliper. The luminal perimeter (Pi) was recorded for the airways of interest. Pi10 was defined as the hypothetical airway with an internal perimeter of 10 mm; thus, the Pi10WA was the wall area of the hypothetical airway with an internal perimeter of 10 mm. All of the airway parameters above (except WA%) were corrected for body surface area (BSA).

CT‐determined pulmonary function parameters, such as LV, MLD, and percent voxels less than −856 HU [VI‐856(%)], were recorded on expiratory phases. The MLD E/I and inspiratory percent voxels less than −950 HU [VI‐950(%)] were calculated. Quantification of CT pulmonary function was performed using whole‐lung densitometry during inspiratory and expiratory CT scans.

### 4.4 Principal Component Analysis

First, the suitability of the data for analysis was assessed by single‐factor analysis. The Kaiser–Mayer–Olkin measure for sampling adequacy was 0.71. Results on the Bartlett test of sphericity reached statistical significance (*p *< 0.0001) [40, 41]. Twelve qCT parameters were analyzed. When two parameters were highly correlated (absolute Spearman rho >0.85), one of them was selected, giving 8 parameters for analysis (Table S1). The variables used were as follows: (1) LA/BSA, (2) WA%, (3) Pi10WA, (4) expiratory LV, (5) expiratory MLD, (6) expiratory VI‐856 (%), (7) mean MLD E/I, and (8) inspiratory VI‐950(%). Before performing the principal component and cluster analyses, all variables were Z‐normalized. We identified three components that contributed to the data set in accordance with the Kaiser criterion (eigenvalue >1) and that accounted for 83% of the total population variance. Component loading for the selected variables of the three independent components is shown in Table S2. Component 1, which accounted for 45% of total variance, correlated with expiratory VI‐856(%), MLD E/I, expiratory LV inversely, and positively with expiratory MLD. Component 2 (24.8% of total variance) correlated with mean WA% and mean Pi10WA, WA% inversely and positively with mean LA/BSA and inspiratory VI‐950(%). Component 3 (14.0% of total variance) inversely correlated with mean LA/BSA and mean Pi10WA and positively with Inspiratory VI‐950(%).

### 4.5 Cluster Analysis

K‐means cluster analysis was performed with the Ward.D method (using Spearman distance as the interval measure) by the R package ConsensusClusterPlus with maxK = 8, 80% item resampling, 50 resamplings, and an agglomerative hierarchical clustering algorithm [42]. The relative change in area under the cumulative distribution function of the consensus index was plotted. The number of likely clusters was determined when there was no appreciable increase. The PAC (proportion of ambiguous clustering) value was used to assess clustering performance. Assessment of the clusterwise stability was performed by the R package fpc with subsetting resampling, and JaccardMean was used to measure the stability of clusters [43].

### 4.6 Conditional Inference Tree Learning

To construct a clinically applicable algorithm for cluster classification, we applied a conditional inference tree analysis to the same clinical variables used in the cluster analysis. This method operates within a conditional inference framework, using binary recursive partitioning to estimate regression relationships [44]. The process involves the following steps: (1) testing the association between each predictor and the response variable, and selecting the most statistically significant one; (2) performing a binary split on the selected predictor at an optimal cutoff point; and (3) recursively repeating this process in the resulting subgroups until no significant associations remain. At each partition, the algorithm identifies the predictor and cutoff value that best split a parent node into two distinct child nodes. The analysis was performed using the “partykit” package in R.

### 4.7 Sputum Collection and Proteomics Analysis

Sputum was induced by the inhalation of hypertonic saline aerosols at increasing concentrations (3%, 4% and 5%). First, the expectorate was collected into a sterile container and processed within 2 h. Second, sputum was isolated from expectoration and treated with four volumes of dithiothreitol for 15 min, followed by four volumes of Dulbecco's PBS solution. Third, the resulting suspension was filtered and centrifuged at 4°C. Finally, the supernatant was aspirated and stored in Eppendorf tubes at −80°C. Additionally, cell smears from the sediment were fixed in neutral formalin and stained with hematoxylin and eosin. Differential cell counts were determined by counting 400 inflammatory cells, while also assessing the quality of the samples. Quality control included viability >75% and squamous cell contamination <30% to minimize saliva or other fluid contamination.

Sputum supernatant samples were subjected to mass spectrometry. We performed the DEPs analysis across the clusters. *p*‐values were adjusted by Benjamini & Hochberg false discovery rate (FDR). For the Reactome pathway enrichment analysis, we obtained the latest gene annotation of Reactome Pathway from Reactome rest API (http://www.reactome.org) [45]. Then, we mapped the DEPs into the background set, and the R software package clusterProfiler (version 4.3.1) was used for enrichment analysis to obtain the protein enrichment. For each signature, we calculated the enrichment scores of the signature in proteomics datasets using GSVA (R package, GSVA) [46]. Cytoscape software and the EnrichmentMap app were used to visualize pathway enrichments as networks [47]. We used the Kruskal–Wallis test for multiple comparisons and Mann–Whitney *U* test for pairwise comparisons of enrichment scores between any two clusters.

### 4.8 Statistical Analysis

The Shapiro test was used to check whether the data were normally distributed, and the Levene method was used to test for multiple‐sample homogeneity of variance. Parametric data are expressed as mean ± standard error of the mean (SEM) and nonparametric data as median (interquartile range [IQR]). Multiple groups were compared using one‐way analysis of variance (ANOVA) (parametric data) or Kruskal–Wallis test (nonparametric data). Pairwise comparison between two groups was tested either by *t*‐test or Kruskal–Wallis test. The chi‐square and Fisher's exact tests were used to compare ratios of category data. R version 4.0.3 (2020‐10‐10) was used for statistical analyses. *p* < 0.05 was considered significant.

## Author Contributions

Qingling Zhang, Meiling Jin, Kian Fan Chung, and Nanshan Zhong conceived of and supervised the project. Zhenan Deng, Tingting Xia, Chenyang Lu, Xuliang Cai, and Yujing Liu analyzed the data and drafted the manuscript. Zhongmin Qiu, Xiaoyang Wei, Wei Gu, Dandan Chen, Jianping Zhao, Xiaoxia Liu, Shenghua Sun, Huaping Tang, Bei He, Shaoxi Cai, and Ping Chen recruited subjects and collected data. All authors have read and approved the final manuscript.

## Funding

This study was supported by the Noncommunicable Chronic Diseases‐National Science and Technology Major Project (2024ZD0529800; 2024ZD0529803), the National Natural Science Foundation of China (82570040; 82400031), the ZHONGNANSHAN MEDICAL FOUDATION OF GUANGDONG PROVINCE (ZNSXS‐20220083; ZNSXS‐20240005), the Guangzhou Municipal Science and Technology Bureau Basic Research Program (2025A03J4397), the grant of State Key Laboratory of Respiratory Disease (SKLRD‐L‐202404; SKLRD‐Z‐202609), the Major clinical research project of Guangzhou Medical University Research Ability Enhancement Program (GMUCR2024‐01010).

## Conflicts of Interest

K. F. C. reports personal fees from attending Advisory Board meetings with GSK, AZ, Novartis, Roche, Merck, Trevi, Rickett‐Beckinson, Nocion & Shionogi. He is a scientific adviser to The Clean Breathing Institute, supported by Haleon, and reports personal fees for speaking at meetings supported by GSK, Sanofi, Novartis & AZ. K. F. C., through his institution, has received research funding from Merck & GSK. Yujing Liu is an employee at AstraZeneca Co., but has no potential relevant financial or nonfinancial interests to disclose. The remaining authors declare no conflicts of interest.

## Ethics Statement

This research was carried out in accordance with the Declaration of Helsinki (as revised in 2013). Written informed consent was obtained from all participants. The study protocol was approved by the Ethics Committee of the First Affiliated Hospital of Guangzhou Medical University (201525003).

## Supporting information




**Supporting Figure S1**: Consensus clustering to classify asthmatics based on qCT parameters. (A) Consensus cumulative distribution function (CDF) of consensus index. (B) Hierarchical structure dendrogram of four clusters identified using Ward's hierarchical clustering. (C) the relative change in area under the CDF curve. (D) The tracking plot showing the allocation of each category to each k (row) sample (column) by color.
**Supporting Figure S2**: Nomogram of ROC curves of different qCT parameters for predicting patients with severe and mild/moderate asthma. ROC, receiver operating characteristic.
**Supporting Figure S3**: Nomogram of ROC curves of different qCT parameters for predicting patients with eosinophilic and non‐eosinophilic asthma. ROC, receiver operating characteristic.
**Supporting Figure S4**: Correlation heat map of sputum proteomics, lung function, and qCT parameters. Red indicates positive correlations, green indicates negative correlations, circle size reflects correlation strength, and “‐” denotes nonsignificant correlations.
**Supporting Table S1**: Spearman correlation between qCT parameters in pairs.
**Supporting Table S2**: Component loading of selected variables.
**Supporting Table S3**: Quantitative CT parameters of the four clusters.
**Supporting Table S4**: Univariate analysis of the relationship between clinical indices and qCT parameters.
**Supporting Table S5**: Molecular pathways obtained from GSVA analysis of sputum supernatant proteomics across clusters.
**Supporting Table S6**: Differentially‐expressed proteins in sputum supernatants across clusters (FC ≥ 1.5, *p*<0.05).

## Data Availability

The raw data that support the findings of this study are available from the corresponding author upon reasonable request.
