# Supplementary material for: Quantitative Computed Tomographic Clusters in C‐BIOPRED Asthma Cohort: Association with Sputum Proteomics
Source: MedComm (2020). 2025 Nov 9;6(11):e70471. doi: 10.1002/mco2.70471 (PMC12596986; doi:10.1002/mco2.70471)
Supplement: Supplementary file 1 — Supporting Figure S1: Consensus clustering to classify asthmatics based on qCT parameters. (A) Consensus cumulative distribution function (CDF) of consensus index. (B) Hierarchical structure dendrogram of four clusters identified using Ward's hierarchical clustering. (C) the relative change in area under the CDF curve. (D) The tracking plot showing the allocation of each category to each k (row) sample (column) by color. Supporting Figure S2: Nomogram of ROC curves of different qCT parameters for predicting patients with severe and mild/moderate asthma. ROC, receiver operating characteristic. Supporting Figure S3: Nomogram of ROC curves of different qCT parameters for predicting patients with eosinophilic and non‐eosinophilic asthma. ROC, receiver operating characteristic. Supporting Figure S4: Correlation heat map of sputum proteomics, lung function, and qCT parameters. Red indicates positive correlations, green indicates negative correlations, circle size reflects correlation strength, and “‐” denotes nonsignificant correlations. Supporting Table S1: Spearman correlation between qCT parameters in pairs. Supporting Table S2: Component loading of selected variables. Supporting Table S3: Quantitative CT parameters of the four clusters. Supporting Table S4: Univariate analysis of the relationship between clinical indices and qCT parameters. Supporting Table S5: Molecular pathways obtained from GSVA analysis of sputum supernatant proteomics across clusters. Supporting Table S6: Differentially‐expressed proteins in sputum supernatants across clusters (FC ≥ 1.5, p<0.05). [file MCO2-6-e70471-s001.docx]

**Quantitative Computed Tomographic Clusters in C-BIOPRED Asthma Cohort: Association with Sputum Proteomics**

Zhenan Deng^1, #^; Tingting Xia^2, #^; Chenyang Lu^1, #^; Xuliang Cai^1, #^; Yujing Liu^3, #^; Zhongmin Qiu^4^; Xiaoyang Wei^5^; Wei Gu^6^; Dandan Chen^7^; Jianping Zhao^8^; Xiaoxia Liu^9^; Shenghua Sun^10^; Huaping Tang^11^; Bei He^12^; Shaoxi Cai^13^; Ping Chen^l4^; Nanshan Zhong^1, 15, *^; Kian Fan Chung^16, *^; Meiling Jin^17, *^ and Qingling Zhang^1, 15, *^, on behalf of the C-BIOPRED Consortium.

^1^ State Key Laboratory of Respiratory Disease, National Clinical Research Center for Respiratory Disease, National Center for Respiratory Medicine, Department of Pulmonary and Critical Care Medicine, Guangzhou Institute of Respiratory Health, The First Affiliated Hospital of Guangzhou Medical University, Guangzhou, China.

^2^ Department of Radiology, The First Affiliated Hospital of Guangzhou Medical University, Guangzhou, China.

^3^ AstraZeneca, Liangjing Rd, Pudong Xinqu, Shanghai, China.

^4^ Department of Pulmonary and Critical Care Medicine, Tongji Hospital, School of Medicine, Tongji University, Shanghai, China

^5^ Department of Respiratory Medicine, The Eighth Medical Center of PLA General Hospital, Beijing, China

^6^ Department of Respiratory Medicine, Nanjing First Hospital, Nanjing Medical University, Nanjing, China

^7^ Department of Pulmonary and Critical Care Medicine, Shenzhen Institute of Respiratory Diseases, The First Affiliated Hospital (Shenzhen People's Hospital) and School of Medicine, Southern University of Science and Technology, Shenzhen, China.

^8^ Department of Respiratory Medicine, Tongji Hospital, Tongji Medical College, Huazhong University of Science and Technology, Wuhan, China

^9^ Department of Respiratory Medicine, Beijing Friendship Hospital, Capital Medical University, Beijing, China

^10^ Department of Respiratory Medicine, The Third Xiangya Hospital of Central South University, Changsha, China

^11^ Department of Respiratory Medicine, Qingdao Municipal Hospital, Qingdao, China ^12^ Department of Respiratory Medicine, Peking University Third Hospital, Beijing, China

^13^ Department of Respiratory Medicine, Nanfang Hospital of Southern Medical University, Guangzhou, China

^14^ Department of Respiratory Medicine, General Hospital of Northern Theater Command, Shenyang, China

^15^ Guangzhou National Laboratory, Bioland, Guangzhou, China.

^16^ National Heart and Lung Institute, Imperial College London, London SW3, UK & Royal Brompton and Harefield Hospital, Guy’s St Thomas NHS Foundation Trust, London SW3, UK

^17^ Department of Respiratory Medicine, Zhongshan Hospital, Shanghai, China

^#^ These authors contributed equally to the article.

^*^ Correspondence to:

Qingling Zhang. Email: zqling@gzhmu.edu.cn.

Meiling Jin. Email: mljin118@163.com.

Kian Fan Chung. Email: f.chung@imperial.ac.uk.

Nanshan Zhong. Email: nanshan@vip.163.com.

**Supplementary files**

Figure S1-S4

Tables S1-S6

C-BIOPRED Consortium members


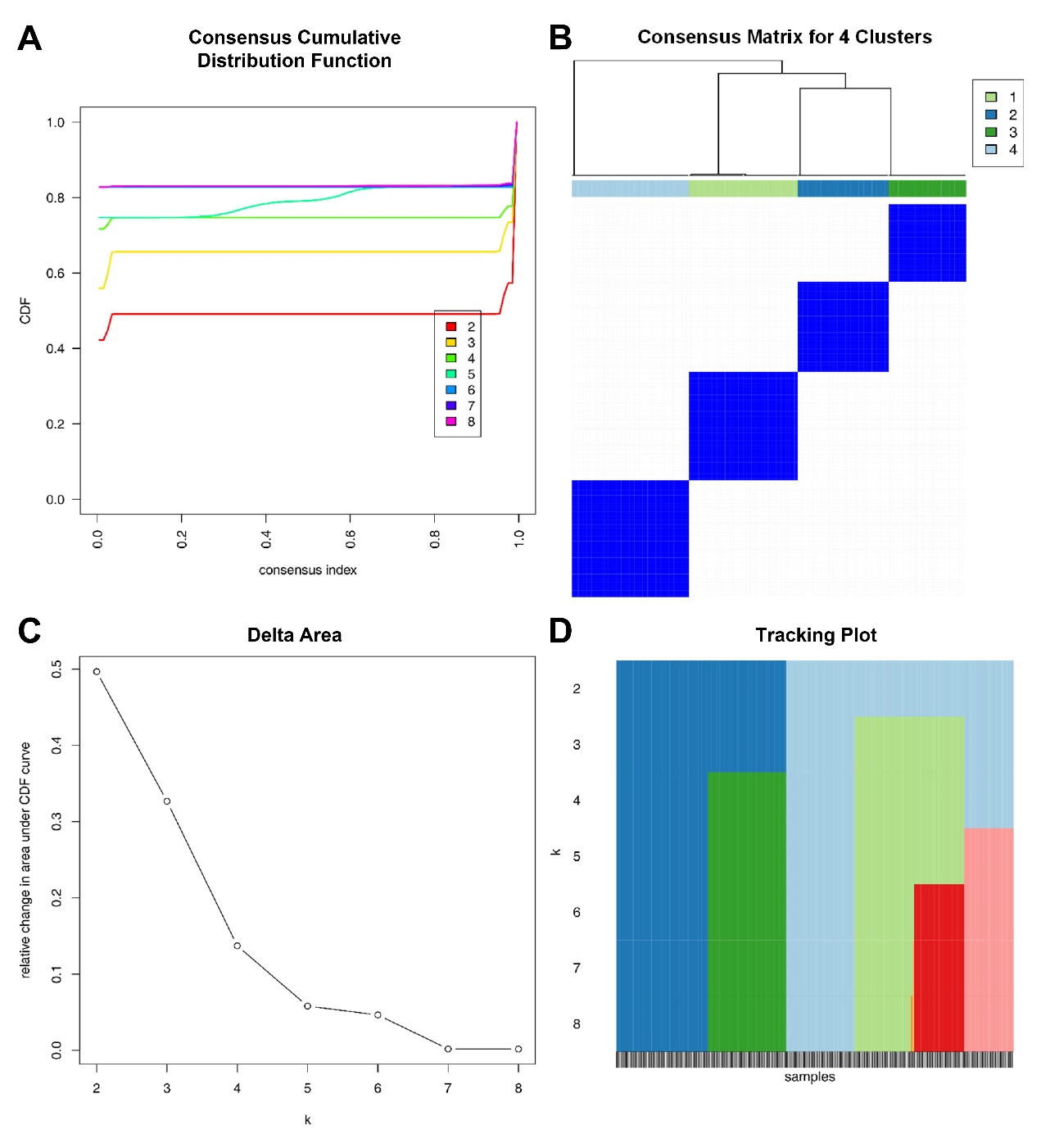


**Figure S1. Consensus Clustering to Classify Asthmatics Based on qCT Parameters.** **A.** Consensus Cumulative Distribution Function (CDF) of consensus index. **B.** Hierarchical structure dendrogram of 4 clusters identified using Ward’s hierarchical clustering. **C.** the relative change in area under the CDF curve. **D.** the Tracking Plot showing the allocation of each category to each k (row) sample (column) by colour.


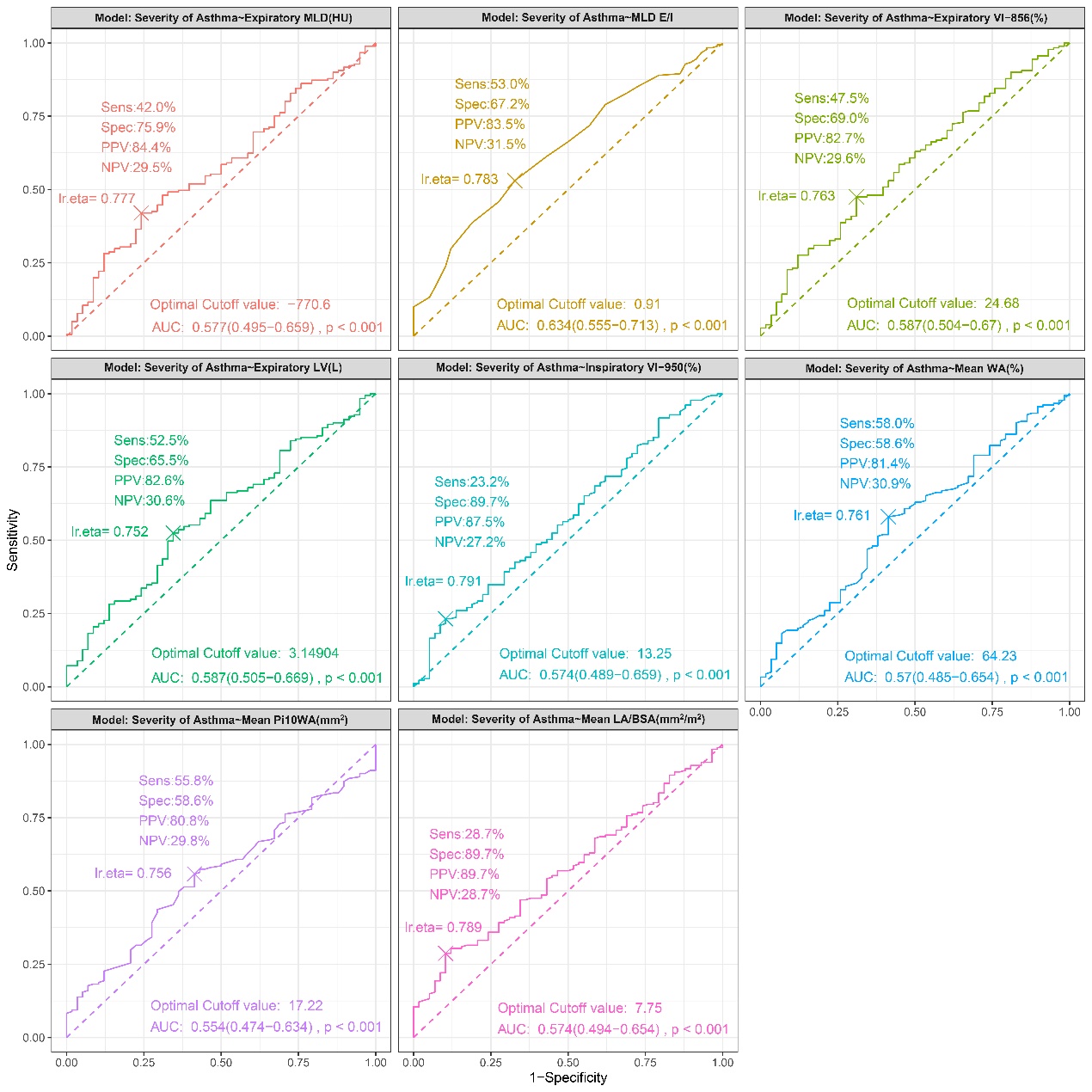


**Figure S2. Nomogram of ROC Curves of Different qCT Parameters for Predicting Patients with Severe and Mild/moderate Asthma.** ROC, receiver operating characteristic.


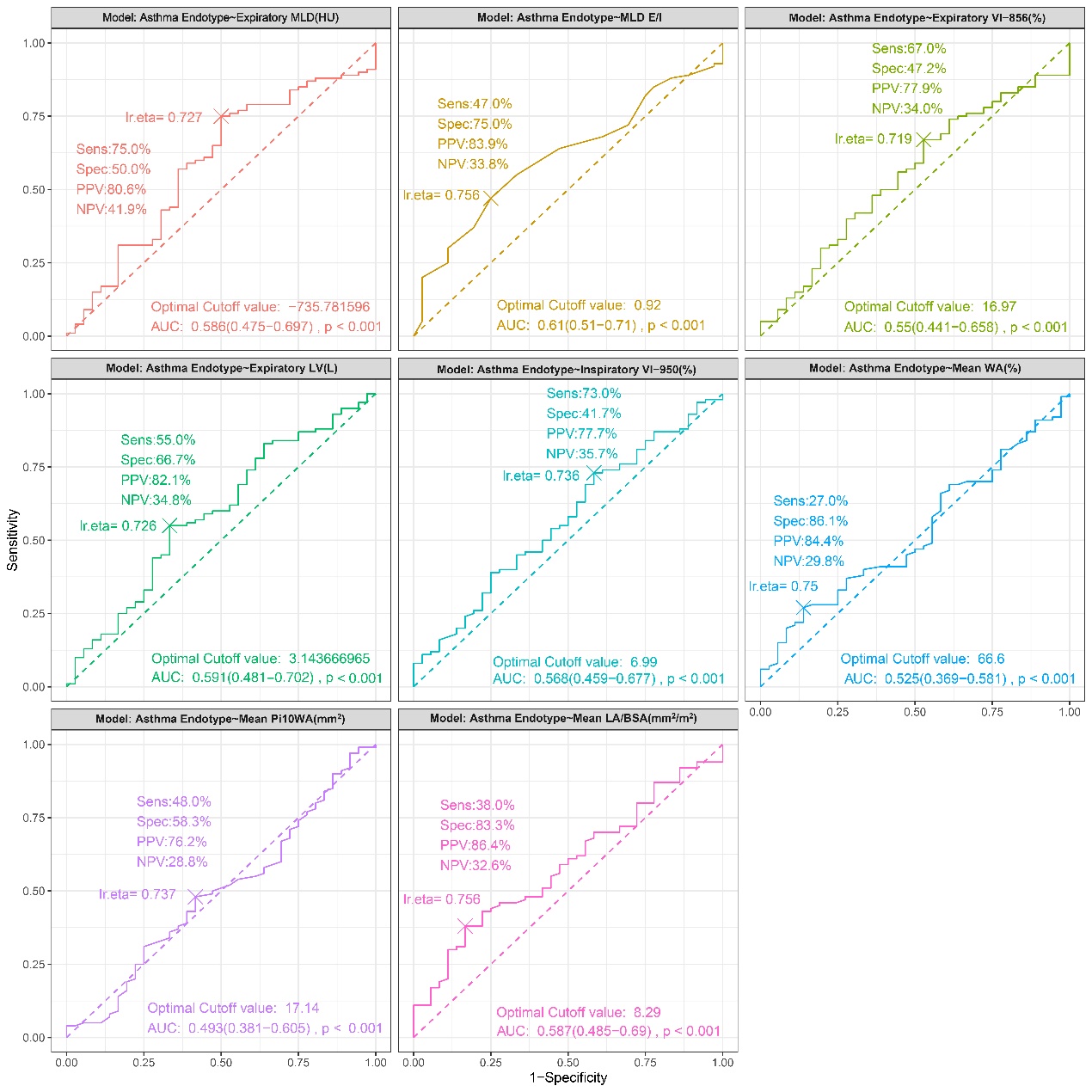


**Figure S3. Nomogram of ROC Curves of Different qCT Parameters for Predicting** **Patients with Eosinophilic and Non-eosinophilic Asthma.** ROC, receiver operating characteristic.


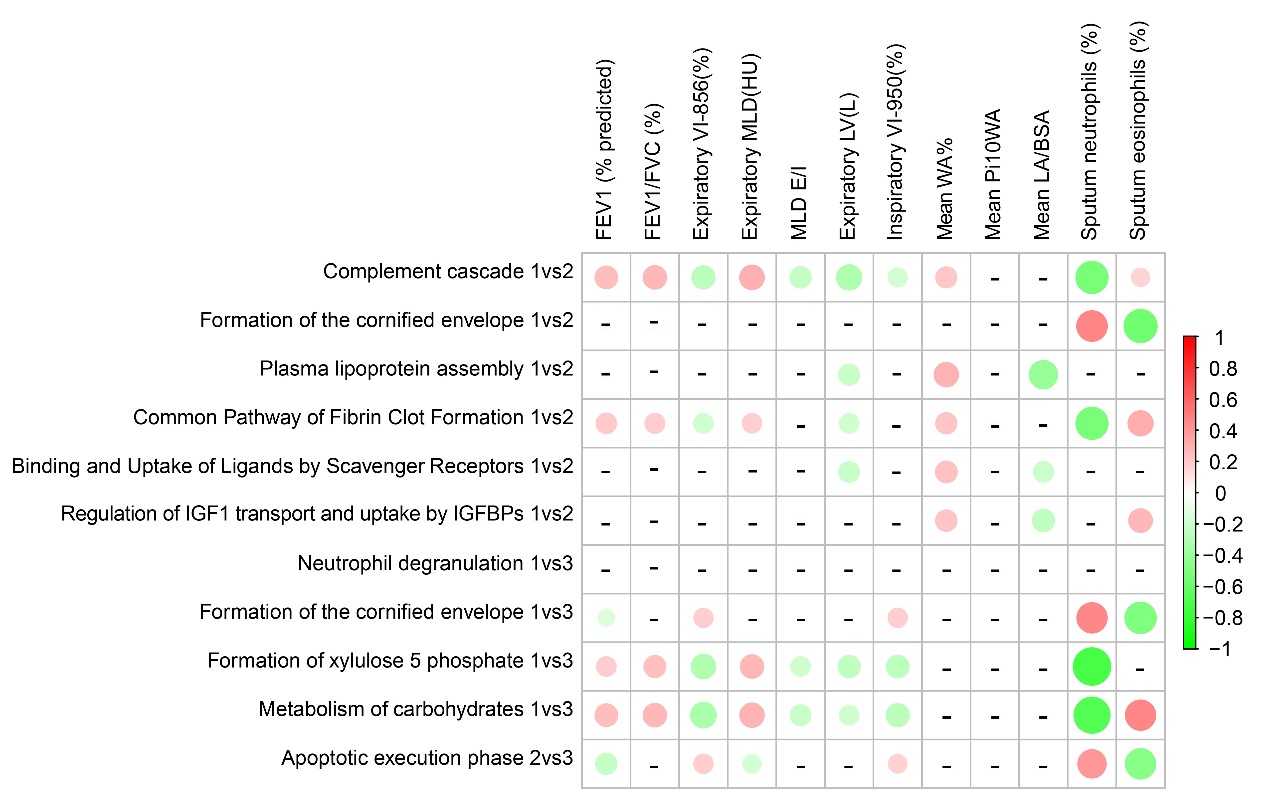


**Figure S4. Correlation Heat Map of Sputum Proteomics, Lung Function, and qCT Parameters.** Red indicates positive correlations, green indicates negative correlations, circle size reflects correlation strength, and "-" denotes non-significant correlations.

| Table S1. **Spearman Correlation between qCT Parameters in Pairs** | | | | | | | | | | | | |
| --- | --- | --- | --- | --- | --- | --- | --- | --- | --- | --- | --- | --- |
| Characteristic | mean LA/BSA | mean WA/BSA | mean WA% | mean Pi10WA | Expiratory LV | Expiratory MLD | Expiratory VI-856(%) | MLD E/I | Expiratory VI-856/-950(%) | VI-856 E-I (%) | VI-856/-950E-I (%) | Inspiratory VI-950(%) |
| Mean LA/BSA | 1** | 0.88** | -0.59** | -0.08 | 0.05 | 0 | -0.03 | -0.08 | 0 | -0.17** | -0.14* | -0.03 |
| Mean WA/BSA | **0.88**** | 1** | -0.17** | 0.2** | 0.07 | 0 | -0.07 | -0.05 | -0.01 | -0.2** | -0.2** | -0.16* |
| Mean WA% | -0.59** | -0.17** | 1** | 0.53** | 0.01 | -0.01 | -0.07 | 0.1 | -0.02 | 0.05 | -0.05 | -0.24** |
| Mean Pi10WA | -0.08 | 0.2** | 0.53** | 1** | 0.12 | 0.04 | -0.12 | 0.04 | -0.05 | -0.01 | -0.11 | -0.32** |
| Expiratory LV | 0.05 | 0.07 | 0.01 | 0.12 | 1** | -0.8** | 0.77** | 0.7** | 0.8** | 0.48** | 0.5** | 0.4** |
| Expiratory MLD | 0 | 0 | -0.01 | 0.04 | -0.8** | 1** | -0.93** | -0.85** | -0.95** | -0.55** | -0.6** | -0.48** |
| Expiratory VI-856(%) | -0.03 | -0.07 | -0.07 | -0.12 | 0.77** | -0.93** | 1** | 0.79** | 0.97** | 0.65** | 0.73** | 0.67** |
| MLD E/I | -0.08 | -0.05 | 0.1 | 0.04 | 0.7** | -0.85** | 0.79** | 1** | 0.8** | 0.77** | 0.74** | 0.25** |
| Expiratory VI-856/-950(%) | 0 | -0.01 | -0.02 | -0.05 | 0.8** | -0.95** | **0.97**** | 0.8** | 1** | 0.58** | 0.65** | 0.57** |
| VI-856 E-I (%) | -0.17** | -0.2** | 0.05 | -0.01 | 0.48** | -0.55** | 0.65** | 0.77** | 0.58** | 1** | 0.96** | 0.29** |
| VI-856/-950E-I (%) | -0.14* | -0.2** | -0.05 | -0.11 | 0.5** | -0.6** | 0.73** | 0.74** | 0.65** | **0.96**** | 1** | 0.49** |
| Inspiratory VI-950(%) | -0.03 | -0.16* | -0.24** | -0.32** | 0.4** | -0.48** | 0.67** | 0.25** | 0.57** | 0.29** | 0.49** | 1** |

| Table S2. Component Loading of Selected Variables | | | | |
| --- | --- | --- | --- | --- |
|  | **Components** | | |  |
|  | 1 | 2 | 3 |  |
| Zscore: mean Expiratory VI-856(%) | -0.504* | -0.017 | 0.067 |  |
| Zscore: mean Expiratory MLD | 0.499* | 0.082 | 0.047 |  |
| Zscore: mean MLD E/I | -0.454* | -0.173 | -0.074 |  |
| Zscore: mean Expiratory LV | -0.439* | -0.114 | -0.244 |  |
| Zscore: Expiratory WA% | 0.060 | -0.647* | 0.169 |  |
| Zscore: Expiratory LA/BSA | -0.019 | 0.421* | -0.716* |  |
| Zscore: Expiratory Pi10WA | 0.071 | -0.502* | -0.472* |  |
| Zscore: Inspiratory VI-950 (%) | -0.298 | 0.321* | 0.405* |  |
| Rotated component matrix | | | |  |
| Extraction method: principal component analysis | | | |  |
| Component loading of the 8 original variables with the 3 main components derived by means of factor analysis in the 239 asthmatic patients is shown, with the predominant variable in each component indicated by an asterisk. | | | |  |

| **Table S3. Quantitative CT Parameters of the 4 Clusters** | | | | | | |
| --- | --- | --- | --- | --- | --- | --- |
| Characteristics | Cluster 1 (n=66) | Cluster 2 (n=55) | Cluster 3 (n=47) | Cluster 4 (n=71) | HC(n=68) | *P* value |
| Mean LA/BSA (mm^2^/m^2^) | 11.66(2.61) | 8.17(1.82) | 10.82(2.75) | 7.82(1.79) | 9.96(3.53) | <0.001 |
| Mean WA% | 61.45(3.32) | 66.00(2.02) | 63.80(1.85) | 66.03(2.47) | 63.72(3.51) | <0.001 |
| Mean Pi10WA (mm^2^) | 15.76(3.14) | 19.36(4.21) | 17.22(2.56) | 17.31(3.84) | 15.92(3.01) | <0.001 |
| Expiratory LV(L) | 3.77(1.71) | 2.65(0.88) | 2.42(0.84) | 3.68(1.71) | 2.57(0.90) | <0.001 |
| Expiratory MLD(HU) | -785.07(48.29) | -722.68(58.96) | -705.90(53.34) | -787.48(58.37) | -721.46(49.47) | <0.001 |
| Expiratory VI-856(%) | 37.40(20.20) | 12.72(12.03) | 10.25(9.89) | 39.70(23.13) | 10.06(13.05) | <0.001 |
| MLD E/I | 0.92(0.07) | 0.88(0.06) | 0.84(0.09) | 0.93(0.05) | 0.87(0.08) | <0.001 |
| Inspiratory VI-950 (%) | 9.18(12.12) | 1.82(3.84) | 1.75(2.63) | 7.98(10.99) | 1.58(2.71) | <0.001 |
| Note: Max: Maximum, Min: Minimum, n: Number of subjects included in the analysis, NA: Not applicable. SD: Standard deviation. Data are shown as median (interquartile range [IQR]), unless as n (%). *P* values were calculated using Kruskal-Wallis Rank Sum Test for continuous variables and Fisher test for categorical variables. | | | | | | |

**Table S4. Univariate Analysis of the Relationship between Clinical Indices and qCT Parameters**

| Characteristic | ACQ5 | AQLQ | FEV1 (% predicted) | FEV1/FVC (%) | FeNO | Sputum eosinophils (%) | Sputum neutrophils (%) | Sputum macrophages (%) | Blood neutrophils (×10^9^/L) | Blood eosinophils (×10^9^/L) |
| --- | --- | --- | --- | --- | --- | --- | --- | --- | --- | --- |
| Mean LA/BSA (mm2/m2) | -0.02 | 0.08 | -0.03 | 0.06 | 0.10 | 0.11 | -0.03 | -0.11 | 0 | -0.13* |
| Mean WA% | 0.053 | -0.021 | -0.196** | -0.116 | -0.046 | -0.05 | 0.066 | -0.022 | 0.025 | 0.059 |
| Mean Pi10WA (mm2) | 0.06 | 0 | -0.05 | -0.09 | -0.09 | -0.17 | 0.23* | -0.08 | 0.01 | -0.02 |
| Expiratory LV(L) | 0.154* | -0.087 | -0.384** | -0.383** | 0.175** | 0.308** | -0.036 | -0.313** | -0.084 | 0.099 |
| Expiratory MLD (HU) | -0.17** | 0.17** | 0.29*** | 0.3*** | -0.18** | -0.24* | -0.12 | 0.48** | 0.03 | -0.03 |
| Expiratory VI-856(%) | 0.17** | -0.19** | -0.25*** | -0.28*** | 0.16* | 0.18 | 0.13 | -0.41** | -0.02 | 0 |
| MLD E/I | 0.19** | -0.18** | -0.24*** | -0.28*** | 0.17* | 0.24* | 0.09 | -0.44** | 0.03 | 0.01 |
| Inspiratory VI-950(%) | 0.14* | -0.2** | -0.12 | -0.12 | 0.04 | 0.04 | 0.11 | -0.2* | -0.02 | -0.01 |

**Table S5. Molecular Pathways Obtained from GSVA Analysis of Sputum Supernatant Proteomics across Clusters.**

| Pathway | C1  median (IQR)  enrichment score | C2  median (IQR)  enrichment score | C3  median (IQR)  enrichment score | C4  median (IQR)  enrichment score | Kruskal Wallis  *P* value | C1 vs C2 *P* value | C1 vs C3 *P* value | C1 vs C4  *P* value | C2 vs C3  *P* value | C2 vs C4  *P* value | C3 vs C4  *P* value | Proteins |
| --- | --- | --- | --- | --- | --- | --- | --- | --- | --- | --- | --- | --- |
| Apoptotic execution phase 2vs3 | 0.39  [-0.33, 0.81] | -0.18  [-0.44,0.55] | -0.38 [-0.84,0.00] | -0.17  [-0.57, 0.50] | 0.013 | 1.00000 | 0.00687 | 0.76510 | 0.15199 | 1.00000 | 0.32646 | H1.4, DSG3 |
| Binding and uptake of ligands by scavenger receptors 1vs2 | -0.25  [-0.64, 0.16] | 0.32  [-0.34, 0.64] | -0.33 [-0.60,0.08] | 0.06  [-0.55, 0.63] | 0.018 | 0.04581 | 1.00000 | 0.61208 | 0.06443 | 1.00000 | 0.64739 | HPR, IGKV1.5, IGLV1.47, APOE, IGHV4.34 |
| Complement cascade 1vs2 | -0.43  [-0.66, 0.30] | 0.34  [-0.26,0.63] | -0.26  [-0.46,0.30] | 0.08  [-0.53,0.53] | 0.004 | 0.00212 | 1.00000 | 0.296218 | 0.11953 | 0.54773 | 1.00000 | IGKV1.5, IGLV1.47, IGHV4.34, PROS1, C8G, C6 |
| Formation of the cornified envelope 1vs2 | 0.24  [-0.15, 0.48] | -0.03  [-0.31, 0.34] | -0.34 [-0.47,0.18] | -0.16  [-0.39, 0.23] | 0.012 | 0.56589 | 0.00819 | 0.14889 | 0.57919 | 1.00000 | 1.00000 | KRT1, CAPNS1, DSP, SPRR2D, SPRR3 |
| Formation of the cornified envelope 1vs3 | 0.31  [-0.17, 0.71] | 0.01  [-0.48,0.49] | -0.46  [-0.58,0.07] | -0.27  [-0.47, 0.47] | 0.005 | 0.53616 | 0.00206 | 0.24349 | 0.26442 | 1.00000 | 0.44894 | KRT1, DSP, DSG3, SPRR1A, DSC2, SPINK5, SPRR3 |
| Formation of fibrin clot 1vs2 | -0.42  [-0.62, 0.00] | 0.39  [-0.40, 0.67] | -0.02 [-0.61,0.21] | -0.02  [-0.52, 0.49] | 0.006 | 0.00394 | 1.00000 | 0.21636 | 0.19694 | 0.98629 | 1.00000 | SERPIND1, PROS1 |
| Formation of xylulose-5-phosphate 1vs3 | -0.30  [-0.58, 0.34] | -0.01  [-0.38,0.47] | 0.39  [0.09, 0.58] | -0.13  [-0.50, 0.35] | 0.002 | 0.28420 | 0.00118 | 1.00000 | 0.34620 | 1.00000 | 0.03464 | AKR1A1, SORD |
| Metabolism of carbohydrates 1vs3 | -0.29  [-0.55, 0.07] | 0.08  [-0.26, 0.40] | 0.27  [0.07,0.48] | -0.09  [-0.46, 0.44] | 0.001 | 0.06902 | 0.00058 | 0.45276 | 0.69139 | 1.00000 | 0.11325 | FBP1, AKR1A1, GOT1, MDH1, SORD, UGP2 |
| Neutrophil degranulation 1vs3 | -0.20  [-0.35, 0.08] | 0.07  [-0.29,0.26] | 0.14  [-0.11,0.33] | 0.01  [-0.25, 0.24] | 0.029 | 0.23289 | 0.02756 | 0.27561 | 1.00000 | 1.00000 | 1.00000 | GMFG, IDH1, CSTB, KRT1, S100A8, TUBB, CXCL1, EEF2, DSP, CDA, MNDA, RAB7A, TUBB4B |
| Plasma lipoprotein assembly 1vs2 | -0.47  [-0.71, 0.09] | 0.22  [-0.27,0.68] | -0.28 [-0.75,0.36] | 0.16  [-0.21, 0.62] | 0.002 | 0.00749 | 1.00000 | 0.02155 | 0.09915 | 1.00000 | 0.22177 | APOE, APOC1 |
| Regulation of IGF transport and uptake by IGFBPs 1vs2 | -0.06  [-0.49, 0.20] | 0.17  [-0.38, 0.60] | -0.43  [-0.59, -0.12] | 0.13  [-0.33, 0.54] | 0.007 | 0.41402 | 0.72038 | 1.00000 | 0.00825 | 1.00000 | 0.03562 | APOA2, SERPIND1 LGALS1, SPARCL1 |

| **Table S6.** **Differentially-Expressed Proteins in Sputum Supernatants across Clusters** (FC≥1.5, P＜0.05) | | | | | | | | | | | | | |
| --- | --- | --- | --- | --- | --- | --- | --- | --- | --- | --- | --- | --- | --- |
| Gene | Protein description | Cluster 1 vs 2 | | Cluster 1 vs 3 | | Cluster 1 vs 4 | | Cluster 2 vs 3 | | Cluster 2 vs 4 | | Cluster 3 vs 4 | |
|  |  | log_2_FC | *P* value | log_2_FC | *P* value | log_2_FC | *P* value | log_2_FC | *P* value | log_2_FC | *P* value | log_2_FC | *P* value |
| TSN | Translin | -1.001 | 0.002 |  |  |  |  |  |  |  |  |  |  |
| SCGB2A1 | Mammaglobin-B |  |  | 1.57 | 0.034 |  |  |  |  |  |  |  |  |
| LYPD3 | Ly6/PLAUR domain-containing protein 3 |  |  | 1.002 | 0.002 | 0.75 | 0.023 |  |  |  |  |  |  |
| S100A8 | Protein S100-A8 | 0.82 | 0.013 | 1.01 | 0.006 |  |  |  |  |  |  |  |  |
| HNRNPA1 | Heterogeneous nuclear ribonucleoprotein A1 |  |  | -1.04 | 0.003 |  |  | -0.79 | 0.022 |  |  |  |  |
| AKR1A1 | Aldo-keto reductase family 1 member A1 | -0.71 | 0.011 | -1.19 | 7e-6 |  |  |  |  |  |  | 0.712 | 0.00 |
| SPRR2D | Small proline-rich protein 2D | 0.94 | 0.026 | 1.32 | 0.019 |  |  |  |  |  |  |  |  |
| SFN | 14-3-3 protein sigma |  |  | 1.12 | 0.004 | 0.64 | 0.041 |  |  |  |  |  |  |
| DSG3 | Desmoglein-3 |  |  | 1.26 | 0.007 |  |  | 0.94 | 0.036 |  |  |  |  |
| SPRR1A | Cornifin-A |  |  | 1.44 | 0.005 |  |  |  |  |  |  |  |  |
| ECM1 | Extracellular matrix protein 1 |  |  | 1.05 | 0.016 |  |  |  |  |  |  |  |  |
| SPINK5 | Serine protease inhibitor Kazal-type 5 |  |  | 1.04 | 0.02 |  |  |  |  |  |  |  |  |
| SPRR3 | Small proline-rich protein 3 | 0.88 | 0.037 | 1.57 | 0.001 | 0.99 | 0.033 |  |  |  |  |  |  |
| KLK1 | Kallikrein-1 | 1.17 | 0.005 | 1.25 | 0.002 |  |  |  |  |  |  |  |  |
| AMY1A | Alpha-amylase 1A | 1.20 | 0.003 | 1.17 | 0.011 |  |  |  |  |  |  |  |  |
| DSP | Desmoplakin | 1.51 | 0.018 | 1.80 | 0.006 |  |  |  |  |  |  |  |  |
| CA6 | Carbonic anhydrase 6 | 1.44 | 0.001 | 1.11 | 0.033 |  |  |  |  |  |  |  |  |
| CST5 | Cystatin-D | 1.46 | 0.001 | 1.37 | 0.004 |  |  |  |  | -0.88 | 0.043 |  |  |
| MUC7 | Mucin-7 | 1.15 | 0.001 | 1.51 | 0.001 | 0.96 | 0.003 |  |  |  |  |  |  |
| BPIFA2 | BPI fold-containing family A member 2 | 1.29 | 0.005 | 1.27 | 0.020 | 0.94 | 0.036 |  |  |  |  |  |  |
| A2ML1 | Alpha-2 macroglobulin-like protein 1 |  |  | 1.54 | 0.003 | 1.28 | 0.010 |  |  |  |  |  |  |
| LCN1 | Lipocalin-1 |  |  | 1.23 | 0.019 | 1.07 | 0.030 |  |  |  |  |  |  |
| TGM3 | Protein-glutamine gamma glutamyltransferase E |  |  | 1.58 | 0.005 | 1.11 | 0.024 |  |  |  |  |  |  |
| MUC2 | Mucin-2 |  |  |  |  |  |  | -1.20 | 0.037 |  |  |  |  |
| SMR3B | Submaxillary gland androgen-regulated protein 3B | 1.41 | 0.046 |  |  |  |  |  |  | -1.41 | 0.041 |  |  |
| CLCA1 | Calcium-activated chloride channel regulator 1 |  |  |  |  |  |  | -2.15 | 0.030 | -2.03 | 0.021 |  |  |
| CST2 | Cystatin-SA | 1.37 | 0.003 | 1.63 | 0.002 |  |  |  |  |  |  | -1.11 | 0.040 |
| CST4 | Cystatin-S | 0.97 | 0.017 | 0.94 | 0.029 |  |  |  |  |  |  |  |  |
| CST1 | Cystatin-SN | 0.90 | 0.018 | 0.86 | 0.041 |  |  |  |  |  |  |  |  |
| IGKV1-5 | Immunoglobulin kappa variable 1-5 | 0.61 | 0.005 |  |  |  |  |  |  |  |  |  |  |
| IGLV1-47 | Immunoglobulin lambda variable 1-47 | 0.82 | 0.002 | 0.68 | 0.013 | 0.63 | 0.017 |  |  |  |  |  |  |
| IGHA2 | Immunoglobulin heavy constant alpha 2 | 0.83 | 0.029 | 0.79 | 0.047 | 0.97 | 0.007 |  |  |  |  |  |  |
| APOE | Apolipoprotein E | -0.77 | 0.002 |  |  |  |  |  |  |  |  |  |  |
| APOC1 | Apolipoprotein C-I | -0.62 | 0.007 |  |  |  |  | 0.69 | 0.020 |  |  | -0.62 | 0.022 |
| LTF | Lactotransferrin | 0.74 | 0.004 |  |  | 0.80 | 0.003 |  |  |  |  |  |  |
| SMR3B | Submaxillary gland androgen-regulated protein 3B | 1.41 | 0.046 |  |  |  |  |  |  |  |  |  |  |
| KRT1 | Keratin, type II cytoskeletal 1 | 0.81 | 0.005 | 0.86 | 0.001 |  |  |  |  |  |  |  |  |
| S100A8 |  | 0.82 | 0.013 | 1.01 | 0.006 |  |  |  |  |  |  |  |  |
| IGHV4-34 | Immunoglobulin heavy variable 4-34 | 0.73 | 0.001 |  |  |  |  |  |  |  |  |  |  |
| KLK1+ | Kallikrein-1 | 1.17 | 0.005 |  |  |  |  |  |  |  |  |  |  |
| PROS1 | Vitamin K-dependent protein S | -0.68 | 0.003 |  |  |  |  |  |  |  |  |  |  |
| ANXA6 | Annexin A6 | 0.71 | 0.046 |  |  |  |  |  |  |  |  |  |  |
| CST2 | Cystatin-SA | 1.37 | 0.003 |  |  |  |  |  |  |  |  |  |  |
| AMY1A | Alpha-amylase 1A | 1.20 | 0.003 |  |  |  |  |  |  |  |  |  |  |
| H1-4 | Histone H1.4 | 0.59 | 0.045 |  |  |  |  | -0.72 | 0.045 | -0.72 | 0.045 |  |  |
| CKB | Creatine kinase | -0.61 | 0.036 |  |  |  |  |  |  |  |  |  |  |
| CDH1 | Cadherin-1 | 0.58 | 0.007 | 0.86 | 0.000 |  |  |  |  |  |  |  |  |
| AKR1A1 | Aldo-keto reductase family 1 member A1 | -0.71 | 0.011 |  |  |  |  |  |  |  |  | 0.712 | 0.00 |
| DSP | Desmoplakin | 1.51 | 0.018 | 1.81 | 0.007 |  |  |  |  |  |  |  |  |
| GOT1 | Aspartate aminotransferase, cytoplasmic | -0.71 | 0.003 |  |  | -0.68 | 0.017 |  |  |  |  |  |  |
| LPO | Lactoperoxidase | 0.61 | 0.005 |  |  |  |  |  |  |  |  |  |  |
| SPRR2D | Small proline-rich protein 2D | 0.94 | 0.026 | 1.33 | 0.020 |  |  |  |  |  |  |  |  |
| CA6 | Carbonic anhydrase 6 | 1.44 | 0.001 |  |  |  |  |  |  |  |  |  |  |
| WARS1 | Tryptophan--tRNA ligase, cytoplasmic | -0.81 | 0.005 | -0.82 | 0.006 | -0.58 | 0.039 |  |  |  |  |  |  |
| STOM | Stomatin OSHomo sapiens | 0.81 | 0.001 |  |  |  |  |  |  | -0.59 | 0.021 |  |  |
| CST5 | Cystatin-D | 1.46 | 0.001 | 1.37 | 0.004 |  |  |  |  | -0.88 | 0.043 |  |  |
| PPP2R1A | Serine/threonine-protein phosphatase 2A 65 kDa regulatory subunit A alpha isoform | -0.59 | 0.013 |  |  |  |  |  |  |  |  |  |  |
| HSPA4 | Heat shock 70 kDa protein 4 | -0.64 | 0.025 | -0.89 | 0.008 | -0.62 | 0.032 |  |  |  |  |  |  |
| MDH1 | Malate dehydrogenase, cytoplasmi | -0.63 | 0.002 | -0.73 | 0.000 |  |  |  |  |  |  |  |  |
| GP2 | Pancreatic secretory granule membrane major glycoprotein GP2 | 0.76 | 0.045 |  |  | 0.87 | 0.009 |  |  |  |  |  |  |
| ARPC4 | Actin-related protein 2/3 complex subunit 4 | 0.68 | 0.026 |  |  |  |  |  |  | -0.64 | 0.036 |  |  |
| PLEC | Plectin | -0.68 | 0.045 |  |  |  |  |  |  |  |  |  |  |
| TSN | Translin | -1.00 | 0.002 |  |  |  |  |  |  |  |  |  |  |
| GOLM1 | Golgi membrane protein 1 | 0.59 | 0.034 |  |  |  |  |  |  |  |  |  |  |
| MUC7 | Mucin-7 | 1.15 | 0.001 | 1.51 | 0.001 | 0.96 | 0.003 |  |  |  |  |  |  |
| ZG16B | Zymogen granule protein 16 homolog B | 0.62 | 0.009 | 0.60 | 0.010 | 0.66 | 0.004 |  |  |  |  |  |  |
| BPIFA2 | BPI fold-containing family A member 2 | 1.29 | 0.005 | 1.27 | 0.020 | 0.94 | 0.036 |  |  |  |  |  |  |
| GLOD4 | Glyoxalase domain-containing protein 4 | -0.84 | 0.007 |  |  |  |  | 0.70 | 0.024 |  |  |  |  |
| MUC5B | Mucin-5B OSHomo sapiens | 0.85 | 0.004 |  |  |  |  |  |  |  |  |  |  |
| SPRR3 | Small proline-rich protein 3 | 0.88 | 0.037 |  |  | 0.99 | 0.033 |  |  |  |  |  |  |
| IGLV3-10 | Immunoglobulin lambda variable 3-10 |  |  |  |  | 0.61 | 0.010 |  |  |  |  |  |  |
| IGHV3-74 | Immunoglobulin heavy variable 3-74 |  |  |  |  | 0.60 | 0.004 |  |  |  |  |  |  |
| IGKV2-29 | Immunoglobulin kappa variable 2-29 |  |  | 0.64 | 0.010 | 0.70 | 0.001 |  |  |  |  |  |  |
| A2ML1 | Alpha-2-macroglobulin-like protein 1 |  |  | 1.54 | 0.003 | 1.28 | 0.010 |  |  |  |  |  |  |
| LYPD3 | Ly6/PLAUR domain-containing protein 3 |  |  |  |  | 0.75 | 0.023 |  |  |  |  |  |  |
| IGKV1D-39 | Immunoglobulin kappa variable 1D-39 |  |  |  |  | 0.58 | 0.044 |  |  |  |  |  |  |
| IGLV1-47 | Immunoglobulin lambda variable 1-47 |  |  |  |  | 0.63 | 0.017 |  |  |  |  |  |  |
| IGHA2 | Immunoglobulin heavy constant alpha 2 |  |  |  |  | 0.97 | 0.007 |  |  |  |  |  |  |
| APOA2 | Apolipoprotein A-II |  |  |  |  | -0.71 | 0.021 |  |  |  |  |  |  |
| LTF | Lactotransferrin |  |  |  |  | 0.80 | 0.003 |  |  |  |  |  |  |
| F5 | Coagulation factor V |  |  |  |  | 0.61 | 0.042 |  |  |  |  |  |  |
| GOT1 | Aspartate aminotransferase, cytoplasmic |  |  |  |  | -0.68 | 0.017 |  |  |  |  |  |  |
| WARS1 | Tryptophan--tRNA ligase, cytoplasmic |  |  |  |  | -0.58 | 0.039 |  |  |  |  |  |  |
| LCN1 | Lipocalin-1 |  |  |  |  | 1.07 | 0.030 |  |  |  |  |  |  |
| SFN | 14-3-3 protein sigma |  |  |  |  | 0.64 | 0.041 |  |  |  |  |  |  |
| STIP1 | Stress-induced-phosphoprotein 1 |  |  | -0.73 | 0.014 | -0.76 | 0.001 |  |  |  |  |  |  |
| HSPA4 | Heat shock 70 kDa protein 4 |  |  |  |  | -0.62 | 0.032 |  |  |  |  |  |  |
| VASP | Vasodilator-stimulated phosphoprotein |  |  | -0.67 | 0.039 | -0.63 | 0.034 |  |  |  |  |  |  |
| HNRNPM | Heterogeneous nuclear ribonucleoprotein M |  |  |  |  | -0.78 | 0.045 |  |  |  |  |  |  |
| CRISP3 | Cysteine-rich secretory protein 3 |  |  | 0.68 | 0.004 | 0.61 | 0.008 |  |  |  |  |  |  |
| GP2 | Pancreatic secretory granule membrane major glycoprotein GP2 |  |  |  |  | 0.87 | 0.009 |  |  |  |  |  |  |
| EIF5A | Eukaryotic translation initiation factor 5A-1 |  |  |  |  | -0.97 | 0.012 |  |  |  |  |  |  |
| TPM4 | Tropomyosin alpha-4 chain OSHomo sapiens |  |  | -0.78 | 0.018 | -0.89 | 0.008 |  |  |  |  |  |  |
| SPTBN1 | Spectrin beta chain, non-erythrocytic 1 |  |  |  |  | -0.83 | 0.049 |  |  |  |  |  |  |
| TGM3 | Protein-glutamine gamma-glutamyltransferase E |  |  |  |  | 1.11 | 0.024 |  |  |  |  |  |  |
| SPTAN1 | Spectrin alpha chain, non-erythrocytic 1 |  |  |  |  | -0.95 | 0.041 |  |  |  |  |  |  |
| ENPP2 | Ectonucleotide pyrophosphatase/phosphodiesterase family member 2 |  |  |  |  | 0.59 | 0.033 |  |  |  |  |  |  |
| HNRNPD | Heterogeneous nuclear ribonucleoprotein D0 |  |  | -0.76 | 0.001 | -0.74 | 0.006 |  |  |  |  |  |  |
| SPARCL1 | SPARC-like protein 1 |  |  |  |  | 0.80 | 0.029 |  |  |  |  |  |  |
| MUC7 | Mucin-7 |  |  |  |  | 0.96 | 0.003 |  |  |  |  |  |  |
| ZG16B | mogen granule protein 16 homolog B |  |  | 0.60 | 0.010 | 0.66 | 0.004 |  |  |  |  |  |  |
| BPIFA2 | BPI fold-containing family A member 2 |  |  |  |  | 0.94 | 0.036 |  |  |  |  |  |  |
| RETN | Resistin |  |  |  |  | 0.70 | 0.028 |  |  |  |  |  |  |
| SPRR3 | Small proline-rich protein 3 |  |  |  |  | 0.99 | 0.033 |  |  |  |  |  |  |
| SERPINB13 | Serpin B13 |  |  |  |  | 0.64 | 0.017 |  |  |  |  | 0.70 | 0.046 |
| PSME2 | Proteasome activator complex subunit 2 |  |  |  |  | -0.82 | 0.010 |  |  |  |  | -0.74 | 0.026 |
| CLCA1 | Calcium-activated chloride channel regulator 1 |  |  |  |  |  |  | -2.15 | 0.030 | -2.03 | 0.021 |  |  |
| APOC1 | Apolipoprotein C-I OSHomo sapiens |  |  |  |  |  |  | 0.69 | 0.020 |  |  |  |  |
| GNAI2 | Guanine nucleotide-binding protein G(i) subunit alpha-2 |  |  |  |  |  |  | -0.67 | 0.007 |  |  |  |  |
| C8G | Complement component C8 gamma chain |  |  |  |  |  |  | 0.80 | 0.035 |  |  | -0.76 | 0.038 |
| FBP1 | Fructose-1,6-bisphosphatase 1 |  |  | -1.00 | 0.004 |  |  | -0.76 | 0.017 |  |  |  |  |
| HNRNPA1 | Heterogeneous nuclear ribonucleoprotein A1 |  |  |  |  |  |  | -0.79 | 0.022 |  |  |  |  |
| H1-4 | Histone H1.4 |  |  |  |  |  |  | -0.72 | 0.045 | -0.72 | 0.045 |  |  |
| HCLS1 | Hematopoietic lineage cell-specific protein |  |  |  |  |  |  | -0.71 | 0.025 |  |  |  |  |
| DSG3 | Desmoglein-3 |  |  | 1.26 | 0.008 |  |  | 0.94 | 0.036 |  |  |  |  |
| MUC2 | Mucin-2 |  |  |  |  |  |  | -1.20 | 0.037 |  |  |  |  |
| PEBP4 | Phosphatidylethanolamine-binding protein 4 |  |  |  |  |  |  | 0.67 | 0.030 |  |  |  |  |
| GLOD4 | Glyoxalase domain-containing protein 4 |  |  |  |  |  |  | 0.70 | 0.024 |  |  |  |  |
| CLCA1 | Calcium-activated chloride channel regulator 1 |  |  |  |  |  |  |  |  | -2.03 | 0.021 |  |  |
| IGHG3 | Immunoglobulin heavy constant gamma 3 |  |  |  |  |  |  |  |  | 0.68 | 0.014 |  |  |
| SMR3B | Submaxillary gland androgen-regulated protein 3B |  |  |  |  |  |  |  |  | -1.41 | 0.041 |  |  |
| H1-4 | Histone H1.4 |  |  |  |  |  |  |  |  | -0.72 | 0.045 |  |  |
| STOM | Stomatin |  |  |  |  |  |  |  |  | -0.59 | 0.021 |  |  |
| CST5 | Cystatin-D |  |  |  |  |  |  |  |  | -0.88 | 0.043 |  |  |
| HNRNPA3 | Heterogeneous nuclear ribonucleoprotein A3 |  |  |  |  |  |  |  |  | -0.59 | 0.005 |  |  |
| ARPC4 | Actin-related protein 2/3 complex subunit 4 |  |  |  |  |  |  |  |  | -0.64 | 0.036 |  |  |
| RACK1 | Receptor of activated protein C kinase 1 |  |  |  |  |  |  |  |  | -0.84 | 0.024 |  |  |
| APOC1 | Apolipoprotein C-I |  |  |  |  |  |  |  |  |  |  | -0.62 | 0.022 |
| ORM1 | Alpha-1-acid glycoprotein 1 |  |  |  |  |  |  |  |  |  |  | -0.60 | 0.007 |
| C8G | Complement component C8 gamma chain |  |  |  |  |  |  |  |  |  |  | -0.76 | 0.038 |
| CST2 | Cystatin-SA |  |  |  |  |  |  |  |  |  |  | -1.11 | 0.040 |
| AKR1A1 |  |  |  |  |  |  |  |  |  |  |  | 0.71 | 0.003 |
| ORM2 | Alpha-1-acid glycoprotein 2 |  |  |  |  |  |  |  |  |  |  | -0.59 | 0.008 |
| AFM | Afamin |  |  |  |  |  |  |  |  |  |  | -0.61 | 0.042 |
| PTGES3 | Prostaglandin E |  |  | -0.70 | 0.011 |  |  |  |  |  |  | 0.70 | 0.012 |
| TMC4 | Transmembrane |  |  |  |  |  |  |  |  |  |  | 0.78 | 0.024 |
| MUC4 | Mucin-4 |  |  |  |  |  |  |  |  |  |  | 0.60 | 0.035 |
| SERPINB13 | Serpin B13 |  |  |  |  |  |  |  |  |  |  | 0.70 | 0.046 |
| PSME2 | Proteasome activator complex subunit 2 |  |  |  |  |  |  |  |  |  |  | -0.74 | 0.026 |
| IGKV3-7 | Probable non-functional immunoglobulin kappa variable 3-7 |  |  | 0.66 | 0.002 |  |  |  |  |  |  |  |  |
| IGKV1-27 | Immunoglobulin kappa variable 1-27 |  |  | 0.62 | 0.004 |  |  |  |  |  |  |  |  |
| IGKV2-29 | Immunoglobulin kappa variable 2-29 |  |  | 0.64 | 0.010 |  |  |  |  |  |  |  |  |
| A2ML1 | Alpha-2-macroglobulin-like protein 1 |  |  | 1.54 | 0.003 |  |  |  |  |  |  |  |  |
| ARPC1B | Actin-related protein 2/3 complex subunit 1B |  |  | -0.62 | 0.002 |  |  |  |  |  |  |  |  |
| ARPC3 | Actin-related protein 2/3 complex subunit 3 |  |  | -0.66 | 0.001 |  |  |  |  |  |  |  |  |
| SCGB2A1 | Mammaglobin-B |  |  | 1.57 | 0.035 |  |  |  |  |  |  |  |  |
| LYPD3 | Ly6/PLAUR domain-containing protein 3 |  |  | 1.00 | 0.003 |  |  |  |  |  |  |  |  |
| CST4 | Cystatin-S |  |  | 0.94 | 0.029 |  |  |  |  |  |  |  |  |
| CST1 | Cystatin-SN |  |  | 0.86 | 0.041 |  |  |  |  |  |  |  |  |
| IGLV1-47 | Immunoglobulin lambda variable 1-47 |  |  | 0.68 | 0.013 |  |  |  |  |  |  |  |  |
| IGHA2 | Immunoglobulin heavy constant alpha 2 |  |  | 0.79 | 0.047 |  |  |  |  |  |  |  |  |
| CSTB | Cystatin-B |  |  | 0.86 | 0.001 |  |  |  |  |  |  |  |  |
| KRT1 | Keratin, type II cytoskeletal 1 |  |  | 0.86 | 0.001 |  |  |  |  |  |  |  |  |
| S100A8 | Protein S100-A8 |  |  | 1.01 | 0.006 |  |  |  |  |  |  |  |  |
| KLK1 | Kallikrein-1 |  |  | 1.26 | 0.002 |  |  |  |  |  |  |  |  |
| DBI | Acyl-CoA-binding protein |  |  | 0.65 | 0.001 |  |  |  |  |  |  |  |  |
| TUBB | Tubulin beta chain |  |  | -0.64 | 0.002 |  |  |  |  |  |  |  |  |
| VIM | Vimentin |  |  | -0.59 | 0.011 |  |  |  |  |  |  |  |  |
| CST2 | Cystatin-SA |  |  | 1.63 | 0.002 |  |  |  |  |  |  |  |  |
| FBP1 | Fructose-1,6-bisphosphatase 1 |  |  | -1.00 | 0.004 |  |  |  |  |  |  |  |  |
| HNRNPA1 | Heterogeneous nuclear ribonucleoprotein A1 |  |  | -1.04 | 0.004 |  |  |  |  |  |  |  |  |
| -- | Immunoglobulin alpha-2 heavy chain |  |  | 0.59 | 0.005 |  |  |  |  |  |  |  |  |
| AMY1A | Alpha-amylase 1A |  |  | 1.17 | 0.012 |  |  |  |  |  |  |  |  |
| CDH1 | Cadherin-1 OSHomo sapiens |  |  | 0.86 | 0.000 |  |  |  |  |  |  |  |  |
| RNH1 | Ribonuclease inhibitor |  |  | -0.73 | 0.000 |  |  |  |  |  |  |  |  |
| EEF2 | Elongation factor 2 |  |  | -0.62 | 0.006 |  |  |  |  |  |  |  |  |
| AKR1A1 | Aldo-keto reductase family 1 member A1 |  |  | -1.19 | 7.4042813398716e-06 |  |  |  |  |  |  |  |  |
| DSP | Desmoplakin |  |  | 1.81 | 0.007 |  |  |  |  |  |  |  |  |
| IL1RN | Interleukin-1 receptor antagonist protein |  |  | 0.68 | 0.020 |  |  |  |  |  |  |  |  |
| TYMP | Thymidine phosphorylase |  |  | -0.66 | 0.004 |  |  |  |  |  |  |  |  |
| TGM2 | Protein-glutamine gamma-glutamyltransferase 2 |  |  | -0.94 | 0.007 |  |  |  |  |  |  |  |  |
| UBA1 | Ubiquitin-like modifier-activating enzyme 1 |  |  | -0.68 | 0.001 |  |  |  |  |  |  |  |  |
| SPRR2D | Small proline-rich protein 2D |  |  | 1.33 | 0.020 |  |  |  |  |  |  |  |  |
| CA6 | Carbonic anhydrase 6 OSHomo sapiens |  |  | 1.11 | 0.033 |  |  |  |  |  |  |  |  |
| WARS1 | Tryptophan--tRNA ligase, cytoplasmic |  |  | -0.82 | 0.006 |  |  |  |  |  |  |  |  |
| CST5 | Cystatin-D OSHomo sapiens |  |  | 1.37 | 0.004 |  |  |  |  |  |  |  |  |
| SERPINB3 | Serpin B3 OSHomo sapiens |  |  | 0.65 | 0.050 |  |  |  |  |  |  |  |  |
| LCN1 | Lipocalin-1 |  |  | 1.23 | 0.019 |  |  |  |  |  |  |  |  |
| SFN | 14-3-3 protein sigma |  |  | 1.12 | 0.004 |  |  |  |  |  |  |  |  |
| STIP1 | Stress-induced-phosphoprotein 1 |  |  | -0.73 | 0.014 |  |  |  |  |  |  |  |  |
| DSG3 | Desmoglein-3 |  |  | 1.26 | 0.008 |  |  |  |  |  |  |  |  |
| HSPA4 | Heat shock 70 kDa protein 4 |  |  | -0.89 | 0.008 |  |  |  |  |  |  |  |  |
| SPRR1A | Cornifin-A |  |  | 1.45 | 0.006 |  |  |  |  |  |  |  |  |
| MDH1 | Malate dehydrogenase, cytoplasmic |  |  | -0.73 | 0.000 |  |  |  |  |  |  |  |  |
| VASP | Vasodilator-stimulated phosphoprotein |  |  | -0.67 | 0.039 |  |  |  |  |  |  |  |  |
| RAB7A | Ras-related protein |  |  | -0.69 | 0.028 |  |  |  |  |  |  |  |  |
| CRISP3 | Cysteine-rich secretory protein 3 |  |  | 0.68 | 0.004 |  |  |  |  |  |  |  |  |
| TPM4 | Tropomyosin alpha-4 chain |  |  | -0.78 | 0.018 | -0.89 | 0.008 |  |  |  |  |  |  |
| DSC2 | Desmocollin-2 |  |  | 0.84 | 0.030 |  |  |  |  |  |  |  |  |
| TGM3 | Protein-glutamine gamma-glutamyltransferase E |  |  | 1.58 | 0.005 |  |  |  |  |  |  |  |  |
| HNRNPD | Heterogeneous nuclear ribonucleoprotein D |  |  | -0.76 | 0.001 |  |  |  |  |  |  |  |  |
| PTGES3 | Prostaglandin E synthase 3 |  |  | -0.70 | 0.011 |  |  |  |  |  |  |  |  |
| CHI3L2 | Chitinase-3-like protein 2 |  |  | 0.90 | 0.003 |  |  |  |  |  |  |  |  |
| ECM1 | Extracellular matrix protein 1 |  |  | 1.05 | 0.017 |  |  |  |  |  |  |  |  |
| UGP2 | UTP--glucose-1-phosphate uridylyltransferase |  |  | -0.81 | 0.009 |  |  |  |  |  |  |  |  |
| SBSN | Suprabasin |  |  | 0.94 | 0.041 |  |  |  |  |  |  |  |  |
| FERMT3 | Fermitin family homolog 3 |  |  | -0.61 | 0.034 |  |  |  |  |  |  |  |  |
| MUC7 | Mucin-7 |  |  | 1.51 | 0.001 |  |  |  |  |  |  |  |  |
| ZG16B | Zymogen granule protein 16 homolog B |  |  | 0.60 | 0.010 |  |  |  |  |  |  |  |  |
| BPIFA2 | BPI fold-containing family A member 2 |  |  | 1.27 | 0.020 |  |  |  |  |  |  |  |  |
| CNDP2 | Cytosolic non-specific dipeptidase |  |  | -0.76 | 0.015 |  |  |  |  |  |  |  |  |
| RNPEP | Aminopeptidase B |  |  | -1.00 | 0.000 |  |  |  |  |  |  |  |  |
| SPINK5 | Serine protease inhibitor Kazal-type 5 |  |  | 1.05 | 0.025 |  |  |  |  |  |  |  |  |
| SPRR3 | Small proline-rich protein 3 |  |  | 1.57 | 0.001 |  |  |  |  |  |  |  |  |

**C-BIOPRED Consortium**

**China-Japan Friendship Hospital:** Jiangtao Lin, Yongming Zhang, Nan Su, Xiaoyan Zhang, Qing Zhao, Ying Nong;

**General Hospital of Northern Theater Command:** Ping Chen, Tianyi Zhu, Binbin Ji, Yan Wang, Haitao Zhao, Jinbao Wang, Zhiyuan Zhang, Xiaona Yang;

**Weifang Asthma hospital:** Chunhua Wei, Jing Han, Nong Yu, Qiang Li, Zhifu Yang, Yanhong Liu, Guohua Li, Huadong Guan, Shuping Zhang;

**The Affiliated Hospital of Inner Mongolia Medical University:** Xiuhua Fu, Lihong Wang, Lingxin Meng, Zhenting Sun, Guangqin Cao, Lei Zhao;

**Beijing Chaoyang Hospital, Capital Medical University:** Kewu Huang, Hong Zhang, Wen Wang, Li An, Yangyu Chen, Yanli Gao;

**Beijing Friendship Hospital, Capital Medical University:** Xiaoxia Liu, Huifen Zhai, Fang Lin, Zhaohui Zhong;

**The First Affiliated Hospital of PLA General Hospital:** Zhongguang Wen, Yan Xiao;

**The First Hospital of China Medical University:** Jian Kang, Lingfei Kong, Xinming Su, Xue Yan, Dijia Zhou;

**Qingdao Municipal Hospital:** Huaping Tang, Wei Han, Yi Shu, Tintian Li;

**Chinese PLA General Hospital:** Lixin Xie, Hong Hu, Bo Liu, Yuzhu Li, Longmei Fan, Yue Zhang, Shifeng Zhao;

**Beijing Tongren Hospital, Capital Medical University:** Xiaofang Liu, Yuhong Wang, Xichun Zhang, Xinmao Wang, Pen Bai;

**Peking University First Hospital:** Guangfa Wang, Yan Hu, Zhanwei Hu, Ju Cao;

**Peking University Third Hospital:** Bei He, Rui Wu, Xiaoyan Gai, Hong Zhu;

**Navy General Hospital:** Zhihai Han, Jiyi Meng, Wei Chen, Li Ma, Zhenqian Liu, Chunyang Zhang, Yan Zhang;

**The Second Affiliated hospital of Zhejiang University School of Medicine:** Huahao Shen, Fugui Yan, Bin Shen, Xinghong Wang, Hao Zhang, Yinghua Yin, Jinkai Liu, Yanxiong Mao;

**Jiangsu Province Hospital:** Mao Huang, Xin Yao, Ji Zhou, Zhenzhen Wu, Ping Yan, Yujie Zhang, Xinling Yang, Ningfei Ji, Fan Fei, Wanzhen Fu, Yi Yang, Yuting Shi, Wangjian Zha;

**Ruijin Hospital, Shanghai Jiao Tong University School of Medicine:** Guochao Shi, Min Zhou, Wei Tang, Wei Chen, Jun Zhou, Wenjie Yang;

**Zhongshan Hospital:** Lei Zhu, Meiling Jin, Liping Xue, Li Li, Chun Li, Yin Gong;

**Shanghai General Hospital:** Qiang Li, Guogang Xie, Luhong Bao, Wuping Bao, Nianyun Li, Fengming Ding, Min Zhang;

**Shanghai Tongji Hospital:** Zhongmin Qiu, Li Yu, Jungang Xie, Lingling Yi, Xianghuai Xu, Qiang Chen, Junjun Tang;

**Shanghai Pulmonary Hospital:** Jinfu Xu, Xiaobing Ji, Jingyun Shi, Bo Su, Xiaojun Yang, Jiuwen Bai, Shuo Liang, Kebing Cheng;

**NanJing First Hospital:** Wei Gu, Jiyong Ma, Yun Liu, Jun Ren, Shan Mao;

**The first affiliated hospital of Guangzhou Medical Hospital:** Qingling Zhang, Baoqing Sun, Jiaxing Xie, Sushan Wei, Rihuang Qiu, Zhiqiang Wang, Peiyan Zheng, Wenting Luo, Changhao Zhong, Wei Luo, Yanqing Xie, Xinxin Yu, Wenting Yu, Wei He;

**Xinqiao Hospital, Army Military Medical University:** Changzheng Wang, Qianli Ma, Min Wan, Mingzhou Zhang, Haining Li;

**Nanfang Hospital, Southern Medical University:** Shaoxi Cai, Haijin Zhao, Yanmei Ye, Fang Zhou, Laiyu Liu, Guohua Huang, Jianpeng Liang;

**Tongji Hospital, Tongji Medical College, Huazhong University of Science and Technology:** Jianping Zhao, Jungang Xie, Qiongjie Hu, Kaiyan Li, Wang Ni, Guohua Zhen, Weining Xiong, Lingling Yi, Xihu Du;

**The First people's Hospital of Yunnan Province:** Yunhui Zhang, Zhi Li, Xiaoqiong Tan, Feng Liao, Shenglan Wang;

**Hainan General Hospital:** Yijiang Huang, Xinjun Cai, Kai Liu, Shaojing Sheng, Yihui Fu;

**The Third Xiangya Hospital of Central South University:** Shenghua Sun, Qiang Zhang, Jinweng Cai, Ye Lin;

**The Second Xiangya Hospital of Central South University:** Ping Chen, Hong Luo, Lv Liu, Zhijun Liu;

**The First hospital of Changsha:** Yuling Tang, Yusheng Yan, Guiyan Mo, Long Wen;

**The Third hospital of Changsha:** Yinqun Zhu, Du Fan.
